# Chemical Precursors of Flocs in Sweetened Beverages: Mechanisms of Formation, Analytical Methods, and Industrial Strategies

**DOI:** 10.3390/molecules31081246

**Published:** 2026-04-09

**Authors:** Ilona Błaszczyk, Radosław Michał Gruska, Magdalena Molska, Alina Kunicka-Styczyńska

**Affiliations:** Department of Sugar Industry and Food Safety Management, Faculty of Biotechnology and Food Sciences, Lodz University of Technology, Wolczanska 171/173, 90-530 Lodz, Poland; radoslaw.gruska@p.lodz.pl (R.M.G.); magdalena.molska@p.lodz.pl (M.M.)

**Keywords:** acid beverage floc, beverage flocculation, alcohol floc, beverage haze, sugar impurities, saponins, protein–polyphenol complexes, haze and turbidity

## Abstract

Flocs, visible particles formed in sugar-sweetened beverages, reduce clarity and consumer acceptance of products. Their presence can be caused not only by different types of trace impurities in the sugar but also by interactions among beverage components. In this review, scientific reports on acid beverage flocs (ABFs) and alcohol flocs are summarized, the main pathways for their formation are described, and practical options for detecting them and preventing their formation in beverages are compiled. Using Preferred Reporting Items for Systematic reviews and Meta-Analyses (PRISMA) 2020 and related guidance, literature searches of Scopus, Web of Science (WoS), PubMed, Food Science and Technology Abstracts (FSTA), CAB Abstracts, and International Commission for Uniform Methods of Sugar Analysis (ICUMSA) resulted in the inclusion of 56 studies. In various types of beverages, complexes formed between proteins (Ps) and polyphenols (PPs) often initiate haze and floc formation, while polysaccharides (dextran, pectin, and starch), silica or silicates, and inorganic ions influence charge balance, particle bridging, and floc growth rate. Ethanol in alcohol beverages can further destabilize colloids and promote aggregation. For beet sugars, saponin–protein interactions are a likely pathway for the formation of ABF, but the available evidence is not consistent. In cane sugars, the reported roles of proteins, polysaccharides, silica, and starch in floc formation vary considerably between studies. For quality assurance, ICUMSA floc tests (GS2-40 and GS2-44) should be complemented by turbidity or haze measurement and colloid characterization such as light scattering, ζ–potential, and infrared IR-based analytical methods supported by chemometrics. Risk mitigation works best as a two-level strategy that combines impurity removal during sugar production and stabilization steps in beverage formulation and storage, including the use of clarification agents and control of pH, temperature, ionic strength, and oxygen exposure. Standardized reporting and validation of rapid predictors against ICUMSA benchmarks remain essential.

## 1. Introduction

Consumer evaluation of food products encompasses several sensory attributes, including color, consistency, and turbidity, as well as the presence of visible undesirable solid particles. Both barely visible specks resembling fibrous fragments and distinct aggregates can reduce product acceptability, as the beverage may be perceived as spoiled or potentially unsafe for consumption. For beverages that are expected to be clear, visual defects may negatively affect consumer perception and lead to economic losses for manufacturers [1]. Rejection of such products may occur solely for esthetic reasons since acid beverage flocs (ABFs) do not pose a health risk [2,3]. For manufacturers of sugar-sweetened drinks and sugar suppliers, the presence of flocs represents a major quality and technological challenge. To describe this visible, undesirable defect in non-alcoholic beverages, the general term “floc” or “flocculated particles” is used, referring to aggregates of suspended particles [4].

The occurrence of this beverage defect can be attributed to the qualities of sugar and water. According to Foong [4], the presence of flocs is associated with interacting sugar impurities that form small aggregates, which may subsequently grow to sizes detectable by visual inspection. Flocs originating from inverted or non-inverted beet or cane sugar are difficult to eliminate and represent a complex technological issue.

Numerical data on the frequency of floc occurrence in finished beverages are rarely published; however, information on the scale of the problem can be inferred from scientific reports addressing the susceptibility of sugar to floc formation. Sastre Siladji et al. [5] found that 15 out of 23 analyzed refined sugar samples were capable of forming flocs based on a 10-day ICUMSA (International Commission for Uniform Methods of Sugar Analysis) GS2-40 (2019) test [6]. Other researchers [7] reported the presence of visually distinguishable flocs in five spoiled fruit-flavored mineral water samples packaged in polyethylene terephthalate (PET) bottles, while ten other samples appeared visually clear or showed only slight turbidity. Coronel et al. [8] and Ruiz et al. [9] reported that approximately 40% of refined sugars and 65% of “común” white sugars were floc-positive in a set of samples from Argentinean and other Latin American producers [10].

Despite decades of research, the mechanisms underlying acid beverage floc formation remain only partially understood, and several important questions have not been answered. The relative contribution of individual sugar impurities to ABF formation is still debated. Studies on cane sugar have attributed the primary role in floc formation to proteins (Ps), starch, silica, or polysaccharides, depending on the source material and analytical approach used. For beet sugar, saponin–protein interactions are the most frequently proposed mechanism, but the evidence is limited and not fully consistent across studies. A further unresolved issue concerns the transferability of findings from model systems to real beverage matrices. Most data have been obtained using acidified sugar solutions prepared under laboratory conditions, and their relevance to commercially formulated beverages has rarely been validated. From an analytical perspective, the ICUMSA GS2-40 test remains the only internationally accepted method for ABF assessment, but its 10-day duration limits its practical utility for real-time quality control. Rapid and quantitative methods capable of predicting the risk of ABFs at the level of individual precursors have not yet been established for routine industrial use. These research gaps motivated this systematic and critical review of the available evidence.

The aim of this review is to provide an integrated systematization and critical evaluation of knowledge on the chemical precursors of flocs introduced via sugar, e.g., polysaccharides, proteins, polyphenols (PPs), and silica, and their roles in the formation of visible flocs in acidic and alcoholic beverages. The review compares floc formation mechanisms, the methods used to detect and characterize flocs and floc active components, and assesses floc formation risk, noting the strengths and limitations of the studies included. It also summarizes technological strategies for reducing sugar impurities and stabilizing beverage formulations during production. The technological strategies described may contribute to the optimization of unit operations during sugar and beverage production to minimize the risk of floc formation in beverages.

## 2. Methods

### 2.1. Reporting Standard

**Figure 1:**
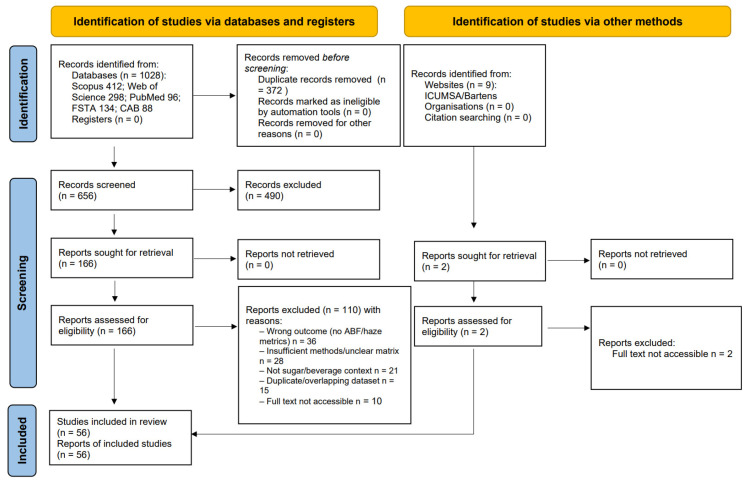
PRISMA 2020 flow diagram for new systematic reviews that include searches of databases, registers and other sources.

This review was prepared in line with PRISMA 2020 (Preferred Reporting Items for Systematic Reviews and Meta-Analyses), using a 27-item checklist and a flow diagram to improve transparency and reproducibility. Scoping components are reported according to Preferred Reporting Items for Systematic Reviews and Meta-Analyses extension for Scoping Reviews (PRISMA-ScR), with 20 essential + 2 optional items, and the search methods and their documentation follow the PRISMA extension for reporting literature searches ((PRISMA-S); 16-item checklist). The PRISMA 2020 flow diagram below was prepared using the official editable template and completed manually with our screening counts. The search strategies, run metadata, and PRISMA counts are presented in Supplement S1 and illustrated by PRISMA 2020 flow diagram (Figure 1) [11,12,13,14].

### 2.2. Eligibility Criteria

Eligibility was defined using the Population, Concept, and Context (PCC) framework for scoping reviews to structure inclusion criteria [15] as follows: Population and context—refined white sugar (beet or cane), liquid sugars, or liquid syrups used in beverages, acidic beverages (typically pH ≤ 4), and alcoholic beverages that use sugar. Concept—acid beverage flocs and alcohol flocs or haze; chemical precursors (plant polysaccharides including dextran, pectin, galactomannans, starch, and β-glucans; proteins and peptides; polyphenols and tannins; silica and silicates); mechanisms (protein–polyphenol interactions; colloidal stability and charge); and analytics, including ICUMSA ABF tests, dynamic light scattering (DLS) and nanoparticle tracking analysis (NTA), and transform infrared (FT-IR) or Fourier transform near-infrared (FT-NIR) spectroscopy. Mitigation options comprised ultrafiltration (UF) and microfiltration (MF), ion-exchange decolorization, polyvinylpolypyrrolidone (PVPP) and silica-gel fining targeting haze-active polyphenols/proteins, bentonite for protein removal, and enzyme aids (e.g., proteases, pectinases). Outcomes—the identification of precursors and mechanisms; ABF results (positive or negative), haze and turbidity, color (IU), % removal, and key process parameters. The studies included were peer-reviewed original studies and reviews, standards and guidelines (ICUMSA), and select conference papers that included methods. ICUMSA GS2-40 [6], GS2-44 [16], and GS2-17 methods were used as definitional anchors for outcomes and terminology [12,17].

### 2.3. Information Sources

The primary databases searched were Scopus, Web of Science (WoS) Core Collection (Science Citation Index Expanded and Emerging Sources Citation Index), and PubMed. To reduce indexing bias in food/beverage research, we also searched Food Science and Technology Abstracts (FSTA) and CAB Abstracts (the CABI database covering applied life sciences, including food science and nutrition). Standards/grey sources included the ICUMSA Methods portal and Bartens trade listings/journals. In this case, “grey” literature comprises research not controlled by commercial academic publishers (e.g., standards, technical reports, and buyers’ guides), consistent with the widely used Luxembourg definition of grey literature [18,19,20,21,22,23,24].

### 2.4. Search Strategy and Search Date

Searches were run on 25 October 2025. Strategies were drafted and peer-reviewed against PRISMA-S. Platform-specific syntax was applied: Scopus TITLE-ABS-KEY with within *n* words, any order (W/*n*) and PRE/*n* (the first term precedes the second within *n* words); Web of Science Topic search (TS) with NEAR/*x* (terms within *x* words; any order). Full line-by-line strategies (with interface, run time, and limits) are provided in Supplement S1. PubMed (Title/Abstract focus): free-text terms for ABF, alcohol floc, haze and protein–polyphenol interactions were combined with sugar terms; date limits used the date of publication [dp]. PubMed help pages guided field/date tagging [25]. Scopus (Advanced): logic equivalent to PubMed and WoS was implemented with W/3 and PRE/n in TITLE-ABS-KEY (W/3 = terms within 3 words, any order; PRE/*n* = the first term must precede the second within *n* words) [26]. Web of Science (Core Collection): TS = topic queries used NEAR/3 proximity as documented by Clarivate (NEAR/*x* = terms within *x* words, any order) [27]. Specialist databases were searched with analogous logic [21,22].

### 2.5. Study Selection and PRISMA Counts

Duplicates were removed before screening using the Bramer field-based sequence (stepwise comparison of key bibliographic fields to detect exact or near-exact matches) [28]. Screening was carried out by two authors in two stages: titles/abstracts were screened first, followed by full texts. Disagreements were discussed and, where necessary, adjudicated by a third author. Screening and recording of reasons for exclusion were managed in Rayyan, using the blinded mode where appropriate [29]. The selection process was reported according to PRISMA 2020; the flow diagram was prepared using the official editable template and completed manually with the review’s counts and is provided in Figure 1. On 25 October 2025, the PRISMA search identified 412 records from Scopus; 298 from Web of Science; 96 from PubMed; 134 from FSTA; 88 from CAB Abstracts; and 9 from ICUMSA (total: 1037). After 372 duplicates were removed, 665 studies remained. Titles/abstracts were screened for 665 (excluding 497); 168 full texts were assessed (112 full texts were excluded with reasons). In total, 56 studies were included in the qualitative synthesis.

## 3. Defining and Classifying Flocs in Sweetened Beverages

Flocs in nonalcoholic beverages, referred to in the beverage and sugar industries as acid beverage flocs, may form in sugar-sweetened, carbonated soft drinks within a few days after manufacture. ABFs suspended in the beverage as visible solid particles may settle at the bottom of the bottle as a sediment layer. These sedimentary flocs appear as tuft-like aggregates or a wispy cloud. Clarke et al. [30] described acid beverage flocs as “a flocculated turbid material” or “cotton ball floc” to denote any form of haze or turbidity in beverages. Haze results from the scattering of light by colloidal or larger suspended particles [31]. Roberts and Godshall [32] suggested that the appearance of flocs depends on the predominant compounds in the beverage. Cane sugars with high starch contents produce cotton-ball-type flocs, whereas those containing silica impurities tend to form granular flocs.

One of the distinguishing features of ABFs is pH-induced aggregation followed by redispersion upon shaking [33]. Uchimiya [34] described ABFs as an example of the Derjaguin–Landau–Verwey–Overbeek (DLVO) theory aggregation of oppositely charged particles occurring under reduced pH.

ABFs may be mistaken for insoluble foreign matter (e.g., hairs, fibers, dust, and carbon particles) introduced with tainted sugar or water or for microbial flocs. Particles of insoluble foreign matter are visible immediately after beverage preparation. Microbial flocs, resulting from contaminating bacteria or fungal development, typically appear after several days as intense turbidity or structures resembling cotton-ball flocs. Such flocs are usually dispersed by vigorous shaking; however, unlike ABFs, they do not disappear completely.

The term alcohol flocs refers to visible, insoluble aggregates of particles that form in alcoholic beverages, appearing as sediment or haze following the addition of ethanol to sugar syrup, beer, or wine [4]. Ethanol induces colloidal destabilization and triggers aggregation.

A universally recognized and formally standardized definition of flocs in beverage systems has not yet been established in the scientific literature, thereby limiting the reproducibility, comparability, and mechanistic interpretation of floc-related phenomena across studies.

## 4. Chemical Impurities in Sugar as Precursors to Floc Formation: History of Floc Research

Various approaches have been employed in the qualitative and quantitative analysis of flocs. Flocs have been produced from commercial beverages, model systems simulating acidic drinks [30], or acidified sugar solutions and solutions of saponins and/or proteins [35]. Sugar impurities regarded as precursors of flocs that form spontaneously in aqueous systems may be carried over from the plant materials, sugar beet root (*Beta vulgaris* L.) or sugarcane (*Saccharum officinarum* L.), used in sugar production. Such compounds may be naturally present in the raw material (e.g., saponins, pectin, polysaccharides, starch, fragments of plant tissues) or may form as a result of chemical transformations or microbial activity during postharvest storage. Floc components may also be generated pending the processing of sugar beet or sugarcane, such as during high-temperature technological operations, through the use of processing aids or as a consequence of microbial growth.

**Figure 2:**
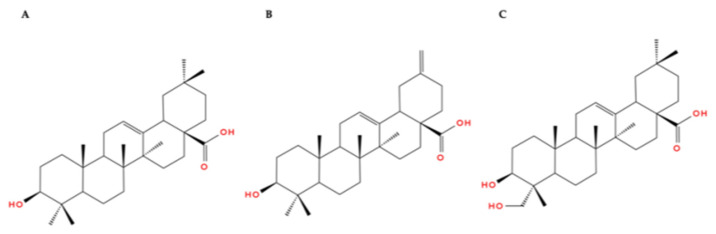
Chemical structures of saponin aglycones present in roots of sugar beet *B. vulgaris* L.: (**A**) oleanolic acid, (**B**) akebonoic acid, and (**C**) hederagenin. Prepared using Molview software v4.10.0.

Research on flocs dates back to the 1950s [36,37]. Since the earliest studies on flocs, the scientific literature has converged on two principal conceptual frameworks for floc formation: discrete sugar-derived impurities [36] or synergistic interactions among multiple constituents within the system [37]. Eis [36] reported flocs isolated from simulated acidic beverages contained saponins and small amounts of high-molecular-weight compounds, which he assumed to be pectins. In turn, Walker and Owens [37] identified a precipitate from an acidified sugar beet solution composed of saponins, fats, and carbohydrates, as well as colloidal carbon and silica particles derived from the sugar-manufacturing process. The authors suggested that saponins act as precursors of floc formation and contribute to the transfer of fats into sugar and ultimately into flocs. They also indicated that silica and colloidal carbon particles did not actively participate in floc formation but were entrapped within floc aggregates. Findings from studies conducted by Roberts et al. [38] in the 1990s likewise failed to demonstrate that saponins are the sole contributors to floc formation. Similarly, Clarke et al. [39] reported that the saponin content in white sugar of standard quality was much lower than the amount required for floc formation; however, they did not specify the threshold level. According to van der Poel et al. [40], the saponin content in white sugar usually does not exceed 0.02 g/kg. The saponins present in sugar originate from sugar beets. Subsequent studies reported saponin concentrations in beet roots ranging from 0.1 to 4.0 g/kg [30,41,42]; however, Edelmann et al. [43] have shown that the saponin content in sugar beet roots falls within the range of 0.862–2.452 g/kg. Typical saponins occurring in sugar beet roots, mainly under the peel [44], include triterpene oligoglycosides [44,45]. Triterpene saponins consist of an aglycone covalently bonded to one or two sugar moieties [46] composed of glucose, xylose, and glucuronic acid [43,46]. Aglycones found in sugar beet roots include oleanolic acid, akebonoic acid, and hederagenin [44,46] (Figure 2). The saponin obtained from sugar beet peel by Morton and Murray [35] incorporated oleanolic acid at a level equivalent to 10% pure saponin.

Despite the contested role of saponins as primary floc precursors, their extracts continued to be utilized in subsequent investigations. Morton and Murray [35] investigated the turbidity of saponin solutions with and without beet sugar protein under neutral and acidic conditions. The significant increase in turbidity of the saponin–protein solution acidified to pH 2 after only 10 min indicated joint involvement of both components in ABF formation. Without agitation, particles exceeding the colloidal size underwent sedimentation [31].

Clarke et al. [30] analyzed the possibility of floc formation in simulated beet or cane sugar beverages, water, and formaldehyde. The addition of oleanolic acid, an oleanolic acid–protein complex, or a methanolic extract of sugar beet to a beet sugar solution could promote floc formation. However, according to Edye [2], the evidence for floc formation in simulated solutions with supplementary components is insufficient to conclude that the mechanism is similar in real beverages.

The evidence supporting saponins as the primary ABF precursor in beet sugar is based predominantly on model experiments using acidified solutions of isolated saponin–protein mixtures or simulated beet sugar beverages, rather than on analyses of commercially produced beverages. These model systems may not accurately represent the complex colloidal environment of a real carbonated soft drink, where interactions among multiple impurities occur simultaneously. As a result, the conclusions drawn from such experiments should be interpreted with caution, and the saponin hypothesis cannot yet be considered definitively established.

There are many studies in the literature focused on identifying floc-prone cane sugars, while comparable data for beet sugars are less abundant. Flocs formed in beverages containing cane sugars may differ in composition from those formed in beverages containing beet sugars.

Flocs forming in beverages sweetened with cane sugar have been studied since 1959, with pioneering studies by Stansbury and Hoffpauir [47,48,49]. They reported that flocs isolated from carbonated soft drinks sweetened with cane sugar were composed of starch, lipids (wax), activated carbon, protein, and ash (mainly silica) [47]. It was initially hypothesized that ABF formation could be initiated by the adsorption of dissolved substances onto carbon particles, which serve as cores for the aggregation of colloidal materials, including starch, silica, proteins, and lipids. Stansbury and Hoffpauir [47] suggested that the amount of flocs formed increased with the amount of carbon used as decolorizing agent in sugar production. In turn, Foong [48] observed larger amounts of cotton-like flocs with increasing silica concentrations. The low pH of beverages was found to be the main driving force behind the colloids involved in floc formation, as many sugar impurities (e.g., proteins) exhibit a low surface charge [47].

Studies on impurities in raw and refined cane sugar and the nature of ABFs have indicated the involvement of proteins, inorganic material (silica), and polysaccharides [50]. Cohen et al. [50] suggested that protein is responsible for initiating floc formation in acidified beverages, as sugars prone to floc formation contained 250 times more protein than those that did not. They suggested that ABFs are formed as a result of interactions between proteins and polysaccharides [4]. Cohen et al. [50] did not observe any correlation between ABF formation and the concentration of inorganic matter or polysaccharides, nor did they find a relationship between the amount of decolorizing carbon used and floc formation. The authors did not confirm the earlier hypothesis proposed by Stansbury and Hoffpauir [47] regarding the role of carbon as an initiator of floc formation. They reported that all sugars prone to ABF formation also produced haze upon the addition of ethanol. However, the sugars that cause alcohol haze do not always exhibit a tendency to form flocs under acidic conditions [50].

In the sugar industry, inorganic impurities (e.g., calcium, silica, and silicate) are collectively referred to as ash. Sugar solutions with a high calcium content show increased turbidity, and the presence of calcium may also lead to the precipitation of other sugar impurities. Silica and/or silicates in sugar and/or in water used for beverage production contribute to haze or floc formation [4].

Roberts and Carpenter [51] found flocs isolated from simulated beverages composed of silica (63.5%), starch (5.5%), monosaccharides (17.7%), proteins (1%), fats, waxes, lipids (5.26%), nitrogen (0.63%), and phosphorus (0.57%). The polysaccharide fraction contained arabinose (0.63%), rhamnose (0.48%), xylose (0.69%), mannose (1.21%), galactose (0.58%), and glucose (14.4%). The presence of these monosaccharides in flocs was confirmed by Miki et al. [52]. Roberts and Carpenter reported amino acids, mostly aspartic acid, alanine, leucine, glutamic acid, serine, threonine, valine, and glycine, identified in floc protein [51]. They [51] also investigated the effect of pH on the rate of floc formation. Flocs formed most rapidly after four days at pH levels of 1.5, 2.0, 3.0, and 4.0. The floc formation time increased by one day at pH 1.0 or by four days at pH 5.0. These findings provided compelling evidence of the pivotal role of beverage pH in governing floc formation. No visible flocs were observed in cane sugar solutions prepared according to the Coca-Cola floc test procedure at pH 6.0 or 7.0 after ten days of storage [51].

Subsequent studies on flocs isolated from beverages containing raw cane sugar included both qualitative and quantitative analyses of floc composition [52,53,54] showing considerable amounts of polysaccharides (23.7–56.4%), silicates (24.8–43.2%), and proteins (5.6–25.7%). The polysaccharide fraction insoluble in water and resistant to acid hydrolysis comprised glucose, mannose, galactose, arabinose, xylose, and rhamnose. Miki [54] revealed that galactomannan takes part in ABF formation in two structures.

Polysaccharides in cane sugar may include dextran and, depending on the cane’s region of origin, levan and sarkaran, starch, the native polysaccharide from sugarcane known as indigenous sugarcane polysaccharide (ISP), and small amounts of cellulose and pectin [2,4]. ISP was first isolated by Roberts et al. [55], who later identified it as an arabinogalactan. Roberts et al. [56] found significant amounts of arabinogalactans when analyzing the polysaccharides in flocs formed in beverages containing sugars expressing varying tendencies to form flocs.

Research conducted over the past decade has confirmed the complex nature of flocs isolated from acidified cane sugar solutions, supporting earlier reports [49]. Lima et al. [49] expanded on previous findings by identifying pectin, phenolic compounds associated with the cell wall (p-hydroxybenzaldehyde and vanillin), as well as fragments of plant tissues (parenchymal cells, xylem, stomata, and epidermis) and lipid substances and starch granules.

Inferences regarding the chemical composition of flocs rarely incorporate statistical analysis [5]. In recent studies, Sastre Siladji et al. [5] found that the effect of starch on floc formation was the most statistically significant when compared to polysaccharides, proteins, and silicates. At the same time, the protein content was found to be, on average, higher in sugars susceptible to floc formation than in sugars in floc-negative samples, although the difference was not statistically significant. Conversely, other researchers have assigned proteins a key role in the mechanism of ABF formation, regarding them as initiators of the process [50].

The views presented in the literature regarding the chemical precursors of flocs formed in the presence of cane sugar remain inconsistent. Sastre Siladji et al. [5] demonstrated that starch had the most significant influence on the process. In contrast, Cohen et al. [50] considered protein to be the initiator of floc formation and to play a crucial role in the process. Roberts and Carpenter [51] reported a high silica content (63.5%) in flocs isolated from simulated beverages. Meanwhile, other researchers [52,53,54] found that flocs isolated from beverages containing raw sugar were characterized by a high content of polysaccharides (23.7–56.4%). Table 1 compares ABF components derived from cane or beet sugar. The variability in floc composition reported across studies highlights the need to define flocs based on their chemical composition and the dominant constituent or group of constituents identified. Although silica-based flocs are described in the literature, classifications such as protein-based, polysaccharide-based, or protein–polysaccharide flocs are not commonly adopted.

Contradictions are difficult to resolve because the studies differ substantially in their design. Some researchers used model solutions prepared from commercial white sugar, while others analyzed authentic flocs isolated from real beverages. The sugar samples examined also originated from different geographical regions and production technologies, which may have resulted in different impurity profiles. Furthermore, the analytical methods used to quantify proteins, polysaccharides, and silica were not uniform across studies, making direct comparison of reported concentrations unreliable. Taken together, the available evidence suggests that no single impurity universally drives ABF formation in cane sugar and that the dominant precursor may vary depending on sugar origin and processing conditions.

## 5. Mechanisms of Floc Formation in Acidic and Alcoholic Beverages

### 5.1. Acidic Beverages: Floc Formation Mechanisms and Factors Affecting the Process

Most studies have focused on identifying the components responsible for the formation of aggregates in acidic or alcoholic beverage sweetened with sucrose. However, there is still limited research on the mechanisms of aggregate formation under different beverage processing and storage conditions [34]. There is only a limited body of literature addressing this topic. A major research gap lies in the lack of a predictive, quantitative, and mechanistically consistent model describing floc formation in acidic beverages.

**Figure 3:**
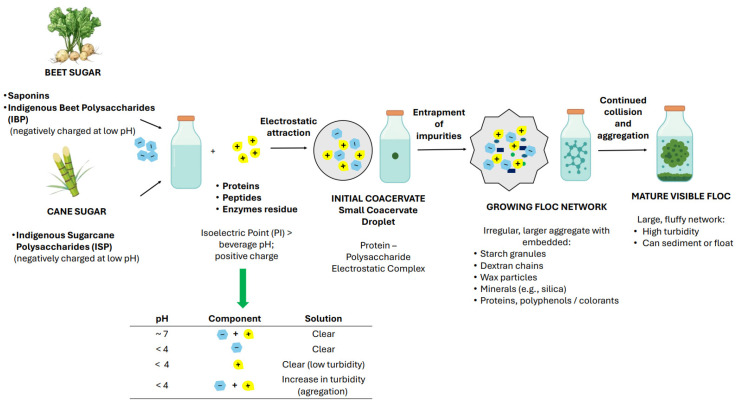
Proposed mechanism of floc formation in sucrose-sweetened acidic beverages. Source: Authors’ work based on [30,35].

Clarke et al. [30] proposed a mechanism for floc formation in beverages sweetened with beet sugar based on the attraction of oppositely charged molecules originating from white sugar. The contributing factors are a negatively charged molecule at a low beverage pH, e.g., saponin (containing a glucuronic acid moiety), oleanolic acid, or polysaccharides derived from the sugar beet cell wall, indigenous beet polysaccharides (IBP) [30], and a positively charged molecule in the acidic environment, such as a protein or peptide with an isoelectric point (pI) above the pH of the beverage. According to Morton and Murray [35], the results of turbidity and interfacial tension measurements suggested the formation of complexes between saponins and proteins in a sugar solution. In their study, the isoelectric pH of the protein was found to be close to 4. At the isoelectric point, proteins are generally the most surface-active due to reduced electrostatic repulsion between molecules (Figure 3). A comprehensive and universally accepted mechanistic model for floc formation in beet sugar solutions incorporating interactions among multiple components—proteins, polysaccharides, fats, and silica—remains to be fully established.

Floc formation in cane sugar solutions is similarly attributed to electrostatic attraction between oppositely charged molecules. In cane sugar solutions, the two factors responsible for floc formation are a polysaccharide containing glucuronic acid and a protein. Initially, an interaction occurs between the indigenous sugarcane polysaccharide (ISP) and a protein. In general, the polysaccharide originates from the plant cell wall, while the protein may be derived from the sugarcane itself or constitute a residue of an added enzyme. Glucuronic acid and primary amine residues carry opposite charges under acidic conditions. At the low pH of the cane-sugar-sweetened beverage, acidic groups are negatively charged, whereas protein groups are positively charged. Molecules associate through electrostatic attraction to form a coacervate, which represents the initial stage of floc development, followed by the formation of a flocculating network that entraps colloidal suspended material, leading to the appearance of visible flocs. Suspended solids, colloids, and high-molecular-weight soluble polymers such as dextran and starch may precipitate from the solution and increase the size of the existing floc, thereby enhancing its appearance [30]. Starch, dextran, waxes, minerals, and proteins involved in floc formation are impurities of cane sugar.

The aggregation of colloids in a solution depends not only on precursors and the pH value but also on ionic strength, temperature, and other external physical factors. Mixing and centrifugation may trigger colloid aggregation, leading to the formation of a more thermodynamically stable agglomerated state [58,59]. Under static conditions, collisions between particles occur primarily as a result of Brownian motion. Agitation increases the frequency of collisions between both individual particles and the forming flocs [4].

A beverage or juice’s pH is a key factor influencing its stability. Changes in pH, combined with the drink’s composition and interactions among components, may lead to distinct aggregation mechanisms [34].

At a neutral pH, no change in turbidity was observed in solutions of beet protein extracts, and saponin, whether examined separately or in combination. Similarly, solutions of saponin or beet protein extracts acidified to pH 2 also showed no detectable change in turbidity. Significant turbidity changes were observed exclusively in the saponin–protein solution at pH 2 [35].

Studies on the influence of various factors on floc formation often focus on sugarcane juice, while beet sugar has received less attention. The colloidal stability and aggregation mechanisms of membrane-filtered sugarcane juice were examined as a function of pH. It was shown in juices with pH below 6.03, aggregation was driven by electrostatic effects, where proteins, polysaccharides, and phenolic compounds interacted with each other, leading to an increase in average aggregate size and turbidity [3]. Sugarcane juice contains a complex mixture of polyphenolic and phenolic flavonoid colorants, which exist in monomeric forms sensitive to pH changes [60]. In an alkaline environment, calcium phosphate flocs adsorb colloidal juice impurities, leading to a rapid increase in turbidity and the formation of sediments [3].

The effects of pH, ionic strength, and temperature in model systems employing an anionic sugar beet extract were investigated. Ralla et al. [45] analyzed the effects of these factors, as well as storage time, on the stability of oil-in-water emulsions with an anionic sugar beet extract. The emulsions remained stable within a pH range of 5–8 and at temperatures up to 60 °C, whereas destabilization was observed at pH 2–4, temperatures above 60 °C, and upon the addition of salts (>0.1 M NaCl or CaCl_2_). It should be noted that emulsions are thermodynamically unstable systems due to the increased interfacial area generated during the emulsification process [61].

Although pH, ionic strength, and temperature are often studied separately, they act jointly, and their effects are interdependent. pH governs the surface charge of colloidal particles and, consequently, modulates the extent of electrostatic attraction between oppositely charged macromolecules: a polysaccharide and protein (cane sugar) or a saponin/oleanolic acid/polysaccharide and a protein/peptide (beet sugar) [30,35]. Ionic strength regulates the range and magnitude of these electrostatic interactions by compressing the electrical double layer around charged particles. Even a modest increase in salt concentration can reduce the repulsive barrier and promote aggregation at pH values at which the beverage would otherwise be stable. Temperature affects both the kinetics and thermodynamics of the process: elevated temperatures accelerate protein unfolding, exposing hydrophobic binding sites and increasing the rate of complex formation, whereas low temperatures slow aggregation kinetics but may promote a reversible chill haze through hydrogen bonding. In practice, these factors rarely vary independently but rather interact in a coupled manner. Therefore, understanding floc formation requires experimental designs that consider these parameters in combination rather than one at a time.

In summary, electrostatic interactions between molecules are crucial in the mechanism of floc formation, and they are strongly influenced by the pH of the beverage.

### 5.2. Mechanism of Haze Formation in Fruit Juices

The ready-to-drink nonalcoholic beverage segment is a diverse product category, including fruit juices, functional beverages (e.g., energy drinks), and flavored water intended for human consumption.

In clear fruit juices, turbidity or sediment may result from microbiological contamination; the degradation of starch, pectins, or tartrates; and the presence of clarifying agent residues. Turbidity in fruit juices is most commonly caused by polysaccharides (i.e., cellulose, hemicelluloses, lignin, starch, pectic substances), proteins, and polyphenols (particularly tannins) [62]. Proteins (P) and polyphenols (PP) can interact to form insoluble complexes through electrostatic and hydrophobic interactions and hydrogen bonding. Polysaccharides can also be associated with polyphenols through hydrophobic, hydrogen, and ionic bonding [62,63].

**Figure 4:**
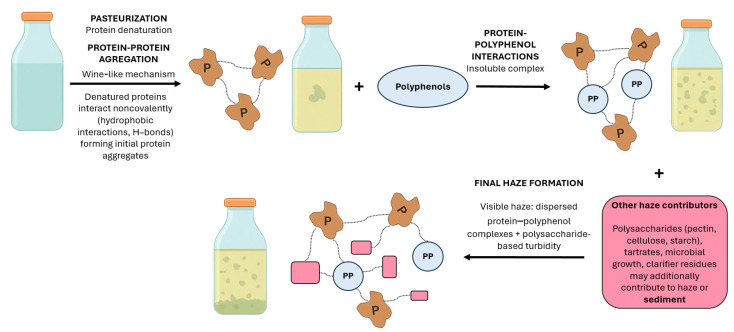
Proposed mechanism of haze formation in fruit juices. Source: Authors’ work based on [62,63,64,65].

The formation of haze in clear apple juices, which are characterized by a relatively low pH, is attributed to proteins originating from the raw material. However, the mechanism of haze formation in clear apple juice has not yet been fully elucidated; it is assumed to be similar to that in white wines, where noncovalent interactions between denatured fruit proteins lead to haze formation. It is believed that the proteins first undergo denaturation and subsequently form aggregates [64]. In apple juice, protein denaturation may occur as a result of pasteurization. The exposure of hydrophobic protein regions facilitates interactions either among protein molecules or between proteins and other juice or wine components, such as polyphenols. Apple juices, particularly those intended for cider production, are characterized by high levels of polyphenols, primarily tannins, which are capable of interacting with proteins and forming aggregates [65]. The proposed mechanism of haze formation pertains to compounds derived from raw materials, without considering the effect of additives such as sucrose [62,63,64,65] (Figure 4). Beet and cane sugar may also contain more reactive denatured proteins involved in floc formation, but no research has been identified in this regard.

Among various possible causes, studies on fruit juice turbidity typically identify protein–polyphenol interactions as the key factor. Fruit juices are stabilized to delay the formation of protein–polyphenol haze. Bentonite efficiently removes proteins and is therefore applied in the stabilization of fruit juices and wine (Siebert, 1999) [66].

### 5.3. Alcohol Flocs: Formation Mechanisms and Determining Factors

**Figure 5:**
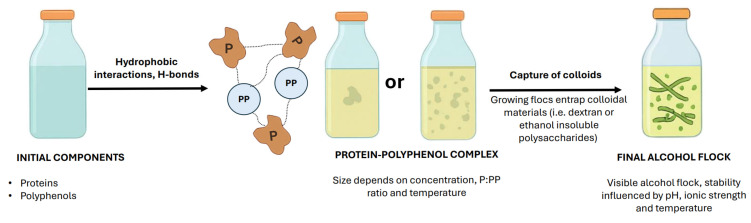
Proposed mechanism of alcohol floc formation in sugar-sweetened alcoholic beverages. Source: Authors’ work based on [60].

The mechanism of floc formation in alcoholic beverages sweetened with cane sugar, as proposed by Eggleston and Triplett [60], is similar to the mechanism in clear apple juice described by Millet [65] in terms of the chemical components that initiate the process. Floc formation occurs in solutions with denatured proteins and polyphenols. Upon reaching a sufficiently large size, they can precipitate together with other colloidal materials, such as dextran and ethanol-insoluble polysaccharides (Figure 5). However, according to Eggleston and Triplett [60], neither insoluble nor soluble starch, fat, inorganic ash, or oligosaccharides, nor parameters such as Brix and pH, played a dominant role in the mechanism of floc formation in alcoholic beverages sweetened with refined cane sugar. In sweetened alcohol beverages, the association of floc components is governed by hydrophobic interactions and hydrogen bonding [60].

Alcoholic beverages, particularly beer produced from barley malt and hops, may develop haze when cooled to temperatures below 5 °C to 0 °C [2,67] due to interactions between proteins and polyphenols [68]. In general, protein–polyphenol haze forms in beer, wine, cider, and fruit juices [69] but is most common in beer [67,70,71,72,73] and wine [74]. Wine, beer, and juices contain polyphenols of various molecular sizes, ranging from very small to considerably larger structures [75]. The size of protein–polyphenol complexes depends on both concentration and the protein-to-polyphenol ratio [76,77]. There are likely critical concentrations of proteins and polyphenols influencing complex formation [60]. The solution pH influences the ionization of proteins and their ionic interactions (either attractive or repulsive), which may alter protein solubility or molecular conformation [69,77] and modify the accessibility of protein binding sites for polyphenols [60]. The brewing industry uses silica to stabilize the product through its interaction with proteins and PVPP to bind polyphenols active in chill and permanent haze [67,77].

Chill haze in beer forms as a result of macromolecules (mainly proteins and polyphenols) associating due to hydrogen bonds between an oxo-group in the protein and hydroxyl groups of the polyphenols. Chill haze is a precursor of permanent haze [67,77], caused by the effect of covalently bonded large molecular substances and preceding hydrophobic interactions and hydrogen bonding [77]. The monomer polyphenols in beer tend to oxidize [67], resulting in their polymerization [78] and the formation of tannins, which subsequently react covalently with proteins, resulting in a permanent haze [79]. Elevated temperatures may lead to the dissolution of loosely bound or newly formed haze, thereby decreasing light scattering [69]. However, studies on model systems have shown that incubation at elevated temperatures leads to stronger protein–polyphenol interactions [80] and increased haze formation [76], probably due to partial protein denaturation that exposes additional polyphenol binding sites.

Scientific studies on wine often focus on the causes of protein haze [64]. Protein haze formation in white wines is associated with the unfolding of proteins upon exposure to elevated temperatures during wine transport or storage. Protein instability may also occur in wines stored properly for more than 12 months [81]. Dufrechou [82] reported that wine pH and ionic strength are key determinants of the aggregation mechanism, aggregate characteristics, and haze formation. The greatest protein instability was observed at 25 °C and pH values below their isoelectric point. At temperatures exceeding 25 °C, haze formation became more pronounced under higher pH values. Furthermore, it was found that the aggregation mechanisms of thaumatin-like proteins and chitinases differ and are influenced by the ionic strength and ionic composition of the wine [64]. Despite the recognized effects of wine pH and salts on protein stability via changes in the ionic strength of the solution, research in this field remains scarce.

Protein–polyphenol interactions can initiate floc formation in both fruit juices and alcoholic beverages through hydrogen bonding and hydrophobic interactions. Modifications in the ionic strength of the beverage and their influence on the stability of macromolecules remain among the less understood factors affecting floc formation in sugar-sweetened beverages.

It should be noted that most studies on protein–polyphenol interactions relevant to alcohol beverage flocculation have been conducted in wine or beer matrices, which differ considerably from sugar-sweetened carbonated soft drinks in terms of pH, alcohol content, and the nature of the proteins and polyphenols involved. Therefore, the direct applicability of these findings to alcohol sugar-sweetened beverages remains uncertain, and studies in relevant beverage matrices are needed.

## 6. Floc Formation Susceptibility Tests and Limitations

The 10-day Coca-Cola floc test [6], based on the observation of a simulated acidic, high-sugar beverage after 10 days, remains the only internationally recognized method of detecting ABFs. The method was initially adopted by the International Commission for Uniform Methods of Sugar Analysis (ICUMSA) in 1970 [83]. The procedure involves dissolving white sugar in water [6], adding formaldehyde in the case of cane sugar [51], and subsequently adjusting the pH to 1.5 with 85% phosphoric acid. The prepared solution is stored for 10 days at room temperature (approximately 25 °C) [51]. There are various modifications of this test, including those extending the observation period to up to 28 days. However, the long time required to obtain results renders the analytical methods used to evaluate the floc-forming tendency of sugar less satisfactory and represents a significant limitation, particularly from the perspective of sugar manufacturers. Modified versions of the official ICUMSA GS2-40 method [6] are being developed, and new approaches that rely on identifying markers of sugar susceptibility to floc formation are being explored.

Another example of a modified Coca-Cola test is the CTC-LA-MT1-025 method (version 4, 14/3/11). The analytical procedure involves dissolving the sugar sample in deionized water, followed by heating, filtration, the addition of sodium benzoate and phosphoric acid, and subsequent dilution with carbonated water. The prepared solutions are stored at room temperature for 10 days and subsequently subjected to visual evaluation [57].

In general, the main differences between the 10-day test and its modifications concern the sample storage time and the application of additional treatments (e.g., heating) and additives (e.g., sodium benzoate).

Ruiz et al. [10] emphasized the need to develop a faster and preferably quantitative test capable of identifying sugar impurities that initiate floc formation in solutions, particularly at a low pH. However, when evaluating alternative ABF indicators for refined sugars, the authors found no correlation between the results of the official 10-day test [6] and either the protein content in the sugar or the absorbance ratio of the sugar solution at pH levels of 9 and 3, findings that contradict those of some earlier studies. McKee et al. [84] analyzed the correlation between the color of refined cane sugar and floc formation in low-pH beverages, finding that refined sugars characterized by a high color index, evaluated as the absorbance ratio of sugar solutions at 420 nm between pH 9 and pH 3, tend to promote floc formation. The color index reflects the presence of naturally occurring polyphenolic and flavonoid pigments in sugarcane.

Carter and Jensen [85] developed a floc-formation susceptibility test for sugar designed to better reflect the composition of commercial acidic clear beverages than the Coca-Cola 10-day floc test [6] or the Spreckels Qualitative Floc Test (24-h Acid Beverage Floc Test for Beet White Sugar), in which a 30% sugar solution, less concentrated than that used in the 10-h test, is acidified to a pH of 2.0 [16]. Carter and Jensen [85] prepared a simulated clear beverage using sugar, demineralized water, citric acid, and sodium benzoate. After transferring the solution into a PET bottle, CO_2_ was added, and the sample was examined for the appearance of flocs after 1, 7, and 10 days. Based on the results, the method was found to be more sensitive than the Coca-Cola 10-day test.

Morton and Murray [35] evaluated the Spreckels test by measuring turbidity spectrophotometrically at 400 nm. The procedure involved preparing a 43% beet sugar solution, acidifying it with orthophosphoric acid to pH 2, and boiling it for 15 min. After cooling, an antimicrobial agent was added, and the solution was visually assessed for haze formation after 24 h. The authors indicated that extending the incubation period (≥48 h) and employing spectrophotometric turbidity measurements could provide a more reliable assessment of the floc potential [35]. After applying a model beverage with raw sugar and small-angle light scattering (SALS), the presence of flocs larger than 100 µm was correlated with a positive outcome in the visual test. Moreover, small-angle light scattering enabled the detection of flocs in the early stages of formation [4].

A key limitation of visual assessment techniques, even those officially approved for evaluating sugar’s floc-forming potential, is their subjective nature, as flocs can be hard to identify due to their small size and low visibility in the solution. Conventional quality parameters, such as color, ash content, and insoluble solids, do not enable the distinction between sugars prone to floc formation and other white sugars. A further limitation concerns the validation of laboratory tests against outcomes in real beverages. Most modifications of the ICUMSA GS2-40 test have been evaluated by comparing their results with this standard test, rather than with floc occurrence in commercially produced beverages under realistic storage conditions. This means that an improved test may be a better predictor than the reference method but not a better predictor of quality deterioration in a real product. Establishing a quantitatively validated relationship between laboratory tests and floc formation in stored commercial beverages remains a critical unmet need in this field.

Producers of alcoholic beverages can evaluate a batch of sugar (e.g., invert sugar) by preparing a 60% alcohol solution. If the solution’s turbidity, expressed in nephelometric turbidity units (NTUs), reaches a value greater than or equal to 2.0 within 30 min, the sugar is classified as unstable. Subsequently, after 24 h, the solution is filtered to remove flocs and used in the production of flavored drinks. Eggleston and Triplett [60] filtered a prepared alcoholic sugar base through diatomaceous earth.

Further research on the analytical evaluation of sugar’s floc-forming tendency requires a comprehensive approach with the application of advanced analytical techniques.

## 7. Analytical Techniques for Floc Characterization and Quantification

The selection of appropriate analytical methods for assessing the susceptibility of sugar to floc formation, along with an understanding of their limitations, is essential. The chemical compounds involved in floc formation may occur in trace concentrations, hindering their detection and quantitative determination. In processed foods, these difficulties are compounded [86]. Various analytical techniques, including microscopy, spectrometry, and chromatography, have been employed to assess the susceptibility of sugars to floc formation.

Fragments of plant tissues such as parenchymal cells, xylem, and stomata were identified by light microscopy in acid beverage flocs isolated from cane sugar solutions [6,49]. Observations using optical microscopy demonstrated the presence of macro-floc structures in sugarcane juices with low levels of non-sucrose impurities [87]. For the qualitative chemical analysis of flocs, scanning electron microscopy (SEM) coupled with energy-dispersive spectroscopic (EDS) analysis was used, which enabled the identification of significant amounts of silicon [49].

Mass spectrometry enables the detection of ionic and ionizable compounds with outstanding speed, sensitivity, and selectivity [88]. The use of high-resolution mass spectrometry by quadrupole time-of-flight (Q-ToF) and matrix-assisted laser desorption/ionization mass spectrometry (MALDI-MS) enabled the identification of triacontanoic acid, hexadecanoic acid, octadecanoic acid, n-octacosanoic acid, and the phenolic compounds *p*-hydroxybenzaldehyde and vanillin in ABFs [49]. The presence of phenolic compounds in brown sugars and sugarcane juice was reported by Payet et al. [89] and Colombo et al. [90], respectively. Payet et al. [89] detected *p*-hydroxybenzoic acid, vanillic acid, homovanillic acid, syringic acid, vanillin, coniferyl alcohol, *p*-coumaric acid, acetosyringone, ferulic acid, and benzoic acid in sugar extracts by liquid chromatography–mass spectrometry (LC-MS). Colombo et al. [90] identified five flavones: luteolin-8-C-glucosyl-7-*O*-glucuronide; tricin-7-*O*-neohesperoside-4′-*O*-rhamnoside; tricin-7-*O*-methylglucuronate-4′-*O*-rhamnoside; tricin-7-*O*-methylglucuronide and swertisin by high-performance liquid chromatography (LC) coupled to diode array ultraviolet (UV) detection and mass spectrometry (MS). The Folin–Ciocalteu assay was used to determine the total phenolic content in brown sugar [89] and sugarcane juice [3]. Eggleston and Triplett [60] noted a strong correlation between the presence of alcohol haze flocs and the color spectrophotometrically measured absorbance at 420 nm.

Trace amounts of protein in refined cane sugars were quantified using ultraviolet–visible (UV-VIS) spectroscopy based on the formation of protein–dye complexes, emphasizing that even trace levels of protein are a key factor in floc formation in carbonated beverages. The dye solution was a mixture of Amido Black 10B (an azo dye), methanol, and glacial acetic acid. Liuzzo and Wong [91] proposed a method for the reliable and rapid prediction of the floc-forming potential of sugar with an absorbance measurement at 630 nm.

Carter [92] developed a protocol for a UV-VIS method for quantifying the protein involved in floc formation by measuring the sugar solution’s absorbance at 230 nm and 260 nm before and after filtration through a laboratory-scale ion exchange membrane adsorber, the Sartobind Q15 (Sartorius, Goettingen, Germany), followed by calculating the protein fraction removed with the flocs. According to Carter [92], this method provides reliable results for protein concentrations between 1.5 and 16 mg/kg in sugar solutions with a dry matter content of 25%. To confirm the key role of protein in forming flocs in the sugar solution, the floc samples were subjected to acid hydrolysis, which enabled the determination of amino acids by high-performance liquid chromatography (HPLC).

Alcohol flocs formed in cane sugar solutions were analyzed using the method applied by the Sugar cane Technology Centre, CTC-LA-MT1-017, version 4, 1/8/11, based on the insolubility of polysaccharides in alcohol. In this approach, the solution turbidity is measured spectrophotometrically at 420 nm, and the results are expressed in absorbance units [57].

According to Lemos et al. [57], the presence of dextran or starch in Brazilian crystalline cane sugar was positively correlated with floc formation in acidic carbonated and alcoholic solutions. Increasing their concentrations resulted in greater absorbance values in the alcoholic floc test (CTC-LA-MT1-017, version 4, 1/8/11). However, Eggleston and Triplett [60] emphasized that the determination of starch in raw sugar according to the ICUMSA GS1-16 (2013) method [93] enables the analysis of soluble starch but does not facilitate the detection of insoluble starch. Consequently, insoluble starch was excluded as a potential component of the flocs. The determination of dextran in sugars by the ICUMSA GS1-15 (2015) method [94] designed for quantitative assessment and applied by Lemos et al. [57] is not highly specific, as it detects not only dextran but also other compounds (e.g., levan) responsible for haze formation [60,95].

In sugar analytics, immunochemical methods are used primarily to detect dextran. Wang et al. [96] developed a specific and sensitive method for rapid, quantitative dextran detection in raw sugar using a sandwich enzyme-linked immunosorbent assay (ELISA) with a detection limit of 3.9 ng/mL. A cross-reactivity test demonstrated the high specificity of the method, with no significant cross-reactions with dextran derivatives. Vanichsriratana et al. [97] developed a test kit for a sandwich ELISA using tetramethylbenzidene (TMB) that was applied in sugar factories for evaluating dextran concentrations. For dextran detection, the enzyme-linked immunosorbent assay demonstrates high accuracy and reliability and requires a short analysis time [97]. According to Wang et al. [96], the use of other methods to measure dextran in sugar and sugar industry by-products is associated with various difficulties. These include, among others, a lack of sensitivity to low-molecular-weight dextran (<10^5^ kDa) and low levels (<0.2 g/kg on solids) when using the enzymatic method, in which the dextran concentration is determined from the difference in haze between dextranase-treated and untreated samples after the addition of ethanol [96,98]; the need for expensive equipment with the enzymatic–chromatographic method combining reversed-phase HPLC with enzymatic hydrolysis [99]; and the high operational costs associated with the optical activity polarimetric method [100]. Alternative tests, often modifications of the alcohol precipitation method employing various chemical and/or enzymatic agents, have also been proposed and evaluated [101,102].

Nephelometry and turbidimetry, both based on light scattering, are well-established and precise measurement techniques used to characterize particle size and size distribution and can be applied in acidic beverages [4]. Turbidimetry relies on the decrease in the intensity of a light beam passing through a sample, while nephelometry measures the intensity of a scattered light beam detected at a 90° angle to the incident beam at 860 nm (near-infrared) (ISO 7027 compliant) [103]. Nephelometry offers highly sensitive direct measurements of insoluble complexes; e.g., protein/tannin aggregates [104]. Eggleston and Triplett [60] reported an increase in light scattering of up to approximately 1000-fold due to the formation of flocs in an alcoholic (60% alcohol by volume (ABV)) solution of invert sugar compared to the turbidity of inverted sugar solutions without ethanol. The result, expressed in NTUs, depends on the number and size of particles in the suspension. Lemos et al. [57] correlated the results of a visual assessment of the floc-forming susceptibility of sugars in a solution and measurable turbidity, expressed in nephelometric units. In this study [57], sugar solutions containing added phosphoric acid were homogenized after 10 days of storage. Turbidity was assessed according to the ratio of reflected and transmitted light. Nephelometric analysis has recently been employed to determine the turbidity of sugarcane juice at various pH values [3]. However, Ahluwalia [105] noted that the turbidimetric method is more appropriate for relatively high concentrations of suspended particles because of extensive light scattering.

Both small-angle scattering (SLS) and dynamic light scattering (photon correlation spectroscopy, PCS) techniques were applied to evaluate particle size as a simple and noninvasive method for monitoring the kinetics of floc growth in simulated acidic beverages with various concentrations of compounds typical of refined sugar: starch, polysaccharides, dextrans, and silica [4]. Foong [4] used light scattering to detect the early stages of ABF formation. According to Uchimiya [34], dynamic light scattering and related colloidal characterization techniques remain underutilized in studies of sugarcane and beet juice.

Laser diffraction (LD) analysis, also known as static light scattering, was used to determine the particle size in sugarcane juice samples [3]. Laser diffraction, together with sieve analysis, is one of the most widely used methods for evaluating particle size distribution.

In the study by Thai [106], the average particle size distribution and fractal dimension of limed sugarcane juice from various sources were evaluated using laser diffraction and dynamic light scattering techniques on the ζ–potential (the zeta potential is the electric potential at the particle’s slipping plane in a liquid and is commonly used as an indicator of colloidal stability). ζ–potential measurements confirmed the negative charge of silica and polysaccharide molecules in solution [4].

To monitor changes in colloid size and charge as a function of pH and ionic strength, atomic force microscopy (AFM) and sedimentation velocity measurements were used in addition to dynamic light scattering and scanning electron microscopy [34]. The X-ray diffraction technique was applied to examine the size and shape of calcium phosphate flocs in synthetic and authentic sugar juices. The authentic sugarcane juice was obtained from sugarcane grown in Australia, whereas the synthetic sugarcane juice samples were prepared from commercial white sugar, water, sucrose, Ca(OH)_2_, KH_2_PO_4_, MgSO_4_·7H_2_0, and *trans*-acontic acid [87].

Understanding the kinetics of heteroaggregation, that is, alterations in colloidal size and charge distribution over time, is essential for predicting quality changes in sugarcane or beet juices resulting from quality deterioration during heat treatment, storage, and transport [34]. Clarke et al. [30] investigated the susceptibility of model solutions to floc formation using beet or cane sugar, water, formaldehyde, oleanolic acid, protein, methanol beet extract, and commercial saponin in various combinations. Thin-layer chromatography (TLC) and gas chromatography–mass spectrometry (GC-MS) were used to identify oleanolic acid and saponins in isolates from beet peel extracts. Other researchers [107] applied a solid-phase-extraction–high-performance liquid chromatography (SPE–HPLC) technique for the direct quantitative determination of triterpenoid saponins in white sugar. The components of purified saponins, hederagenin, akebonoic acid, and oleanolic acid, were identified using liquid chromatography–mass spectrometry (LC-MS) [44]. Gas–liquid chromatography was employed to analyze the composition of the polysaccharide fraction isolated from flocs formed in simulated acidic beverages prepared according to the Coca-Cola floc test procedure [51]. Eggleston and Triplett [60] used ion chromatography with pulsed amperometric detection (IC-PAD) to determine oligosaccharides in alcoholic beverages sweetened with cane sugar.

Despite its complexity, stemming from the numerous possible combinations of modules, columns, mobile phases, and parameters, HPLC remains the preferable analytical technique owing to its versatility, reproducibility, and broad applicability. The greatest advantage of this quantitative, sensitive, and highly precise method lies in its ability to analyze a broad spectrum of analytes, ranging from small organic molecules and ions to large biomolecules and polymers [88].

HPLC was used for the analysis of amino acids from floc samples following acid hydrolysis. The flocs were isolated by dialysis and centrifugation after adjusting the pH of the sugar solutions to 3 [92]. The free amino acid content in sugarcane juice was determined using an automatic amino acid analyzer, following the method described by Adhikari et al. [108].

In a recent study, Sastre Siladji et al. [5] examined the correlation between the results of the standard 10-day test and the contents of selected components, including starch, proteins, polysaccharides, and silicates. The analyses were carried out according to the ICUMSA GS1-17 [109] method (starch determination, spectrophotometric method), Bradford method (protein determination, spectrophotometric method) [110], a modified Roberts method (polysaccharide determination) [111], and the colorimetric heptamolybdate method for water analysis [112], which differentiates reactive silicates at room temperature from polymeric or colloidal silicates that become reactive upon heating [113].

The impact of impurities on the physicochemical characteristics of calcium phosphate flocs formed during sugarcane juice clarification was examined using the following instrumental techniques [114]: small-angle laser light scattering, attenuated total reflectance Fourier transform infrared spectroscopy (ATR-FTIR), and X-ray powder diffraction (XRD). Fourier transform infrared (FTIR) spectroscopy was employed to analyze aggregates in the freeze-dried powder of sugarcane juice [3]. In turn, the ζ–potential measurements of particles generated by different liming techniques were used to elucidate differences in clarification efficiency [115].

Molecular-level changes can be detected much more rapidly by spectrophotometric methods [35] than by visual changes in the solution. Further research is needed to establish a correlation between spectrophotometrically detected changes and the formation of flocs visible to the naked eye. Results obtained from model systems should be verified in situ in beverages.

Although various techniques have been employed to analyze flocs formed in sugar-containing liquids, there is still a lack of studies addressing the application of near-infrared (NIR) spectroscopy. Due to its rapid measurement capability and minimal sample preparation requirements, this technique may serve as a valuable tool for evaluating the chemical composition and properties of flocs. Moreover, NIR spectroscopy can complement other analytical techniques, e.g., DLS, FTIR, and HPLC, to provide a more comprehensive characterization of the flocculation phenomenon.

The methods described in this section differ substantially in their detection range, chemical specificity, and suitability for routine or research applications. Table 2 compares the main analytical approaches used in ABF research; an expanded version with detailed sensitivity data and recommended use cases is provided in Supplementary Table S1.

As shown in Table 2, no single method is sufficient to provide a comprehensive assessment of floc-forming potential. The visual ICUMSA GS2-40 test for sugar flocculation propensity remains the key reference endpoint for ABF risk classification. However, the results of this test provide limited mechanistic insight and low sensitivity to early-stage colloidal aggregation. Colorimetric assays and UV-Vis spectrophotometry enable cost-effective screening of individual precursor classes, but their selectivity is limited, and their predictive value depends on calibration against a reference floc endpoint. Light scattering techniques and ζ–potential measurements are currently underutilized in ABF research despite their sensitivity to pre-visual aggregation. The few studies that have applied these methods to sugar-relevant systems demonstrate their potential for the early detection of colloidal instability, but the available data are insufficient to establish validated decision thresholds for industrial use. Systematic studies correlating DLS-derived aggregation kinetics with ICUMSA GS2-40 outcomes across a range of sugar types and impurity levels are needed before these techniques can be recommended for routine quality control. Chromatographic and mass spectrometric methods offer the highest chemical resolution and are particularly effective for confirmatory analysis and the resolution of competing hypotheses concerning precursor identity. For routine industrial use, colorimetric assays and ICUMSA tests remain the most practical choices. In studies focused on the elucidation of specific precursors or resolving mechanistic questions, chromatographic and mass spectrometric methods are preferred. Light scattering and ζ–potential measurements are recommended when determining timing and the mechanism of aggregation is the overarching objective. NIR spectroscopy combined with chemometrics represents a promising direction for at-line screening but requires validation against ICUMSA benchmarks before broader application in sugar production. Further methodological details are provided in Supplementary Table S1.

Minimizing acid beverage floc formation in sugar-sweetened beverages requires a comprehensive, multi-level strategy combining actions at both the sugar production and beverage formulation stages. The effective reduction in sugar impurities through the selection of raw materials, optimized clarification and filtration processes, and the application of sustainable and innovative purification methods and simultaneous use of strategic use of flocculation inhibitors, biopolymers, electrostatic stabilizers, advanced adsorbents, and emerging enzymatic treatments can further enhance beverage stability by disrupting or preventing aggregation phenomena. Finally, precise control over beverage production processes, including pH, temperature, real-time analytical monitoring, and raw material selection are essential tools for ensuring beverage clarity and consumer acceptance. Holistic integration of these strategies enables manufacturers to meet consumers’ quality demands.

## 8. Strategies for Mitigating Floc Formation in Beverages

### 8.1. Raw Material Quality Control (QC) and Impurity Reduction in Sugar Production Technologies

Chemical impurities in sugar play a crucial role in the formation of flocs in sugar-sweetened beverages. Several technologies can be implemented to minimize impurities at the sugar production stage, which is essential for reducing the risk of visible defects in the final product.

The basic strategy is restrictive quality control of raw material to limit the initial load of potential floc precursors. In practice, both visual grading and analytical monitoring of incoming sugar beet and cane should be conducted [49,116,117]. At the reception stage, roots and stalks should be assessed for extraneous matter (soil, stones, tops, and leaves), visible deterioration or microbial spoilage, and the crown/top ratio. The tissues originating from plant crowns and tops contain more non-sugars and cell wall material than the storage root or stalk internodes. Routine analytical checks should also cover key molasses-forming non-sugars such as potassium, sodium and α-amino nitrogen, invert sugars and, where relevant, dextran or other microbial polysaccharides, as these parameters are strongly associated with poorer processing quality and higher carry-over of plant polysaccharides, proteins, and silica into the sugar raw juice and, ultimately, into white sugar, increasing the ABF risk.

Another strategy focuses on optimizing juice clarification and filtration. Clarification is fundamental for removing colloidal impurities. In both cane and beet sugar manufacturing, lime is added, followed by carbon dioxide, which precipitates calcium carbonate and helps capture impurities. By contrast, phosphatation uses a combination of lime and phosphoric acid to generate calcium phosphate flocs that remove impurities. The available evidence indicates that carbonatation can be more effective than phosphatation in reducing floc precursors [118,119]. Moreover, fine filtration through materials such as celite or kieselguhr at temperatures below 60 °C significantly reduces suspended solids that may form acid beverage flocs. Filtering sweet syrups at lower temperatures has been reported to prevent floc formation, although the underlying mechanisms require further investigation [119]. Additionally, recent studies indicate that membrane-based clarification methods, particularly ultrafiltration (UF) and microfiltration (MF), are effective alternatives to traditional clarification processes. Ultrafiltration using ceramic or stainless-steel membranes has enabled significant reductions in turbidity, color, and suspended macromolecular impurities, directly minimizing the precursors responsible for ABF formation. Moreover, membrane-based processes offer additional environmental benefits by eliminating the need for chemical flocculants and filtration aids [120,121].

The next key strategy involves targeting specific impurities contributing to floc formation, such as proteins, polyphenols, residual cellulosic materials, and polysaccharides, using filtration, adsorption, or chemical clarification techniques [120,121,122,123,124]. In practice, pressure-driven membranes (UF/MF) with pore sizes in the nano- and microfiltration ranges can selectively retain high-molar-mass impurities (proteins, polysaccharides, and fine cell wall fragments) while allowing sugars and salts to pass, which markedly lowers turbidity and color in limed sugarcane juice and clarified cane juice [120,121]. Clarification aids such as lime, bentonite, and activated carbon are applied to agglomerate or adsorb suspended particles and colored colloids before syrup concentration, further reducing haze-active materials [122]. For more specific removal of colored and charged compounds, adsorptive resins (e.g., anionic ion-exchange resins) can decolorize sugar solutions by binding high-molecular-weight colorants and associated ions [123]. Finally, enzymatic or chemical clarification, such as protease-assisted treatment combined with gelatin/silica or other fining agents, can partially hydrolyze and precipitate protein–polyphenol complexes, thereby decreasing both haze and phenolic load in juice-type matrices [124]. Calcium addition promotes the precipitation of negatively charged impurities, such as pectic and other acidic polysaccharides, protein–polyphenol complexes with a net negative charge, and inorganic anions (e.g., phosphate, sulfate, silicate), enabling their subsequent removal through filtration [119]. Adjusting the ionic conditions, pH, and adsorption steps is therefore essential to minimizing ABF risk [118]. Beyond conventional clarification, filtration and ion exchange approaches, recent work has also focused on more sustainable purification concepts: the use of bio-based flocculants derived from modified cellulose extracted from sugarcane bagasse. These natural, biodegradable, anionic polyelectrolytes have demonstrated effectiveness comparable to that of synthetic polymers such as polyacrylamide while offering advantages in terms of biodegradability, non-toxicity, and alignment with circular bioeconomy principles [125].

Another effective approach to reducing ABF precursors involves ion-exchange treatments. Recent developments have shown that mixed-bed ion-exchange resins (weak-base/strong-acid types) efficiently remove residual ionic and charged colloidal impurities, including polysaccharides, proteins, calcium, and magnesium salts. Such treatments notably enhance beverage clarity and stability, significantly mitigating the risk of ABF formation. A recent application of these methods achieved considerable ion reductions and complete elimination of floc formation in treated cane syrup, further underscoring their practical applicability [123,126,127,128].

Effective, substantial reductions in sugar impurities require an integrated approach combining technological improvements in sugar processing with careful raw material selection (sugar beets with good technological value) to meet the quality demands of the beverage industry.

### 8.2. Application of Flocculation Inhibitors

A promising approach to minimizing ABF formation is to use inhibitors to prevent or disrupt the aggregation processes. This strategy can be applied both at the sugar-processing stage and during beverage formulation.

Recent research highlights the potential of bio-based polymers, such as modified cellulose derivatives, to act not only as flocculants in sugar clarification but also as stabilizers that prevent undesirable aggregation in beverages [125,129,130,131]. These natural polyelectrolytes, derived from sugarcane bagasse or other agricultural by-products, are biodegradable, non-toxic, and align with the principles of the circular bioeconomy. By modifying surface charge interactions or by steric stabilization, creating strong repulsion between particles and droplets in a dispersion [132], natural polyelectrolytes can reduce the likelihood of coacervate formation between oppositely charged particles.

To prevent ABF formation, electrostatic stabilization by reducing the ionic strength of the drink, by limiting added salts or using gentle buffering agents like citrate or phosphate to keep the pH stable, is recommended [66,133]. Calcium or magnesium ion contents should be kept low or carefully controlled, as they may bridge negatively charged chains and promote aggregate formation [134,135,136].

A further strategy involves the use of agents that block polyphenol–protein interactions in alcoholic beverages. The strategy, used in brewing, of preventing haze formation by adding silica hydrogels or tannic acid binding agents may be adapted to sugar-sweetened alcoholic beverages to inhibit similar flocculation phenomena [31,67,137]. These inhibitors act by either binding to the reactive sites of polyphenols or adsorbing haze-forming proteins. Other research highlights the effectiveness of chemically modified bentonites and hybrid PVPP silica adsorbents in managing protein–polyphenol haze. These novel fining agents exhibit superior binding affinity toward proline-rich proteins and polyphenolic haze precursors compared to conventional bentonite. Modified calcium–bentonite demonstrated significant haze-active protein removal at substantially lower dosages, preserving beverage aroma and sensory characteristics. This technological advancement is promising for the mitigation of ABF-related haze formation in beverages [122,138,139,140,141,142,143,144].

An alternative approach comprises enzymatic treatments. Although not widely adopted in the sugar or soft drink industries due to process limitations. Proteolytic enzymes can theoretically degrade the macromolecular precursors of acid beverage flocs, preventing their interaction. Recent advances in juice clarification technologies suggest that pectinases, cellulases, β-glucanases, and proteases can improve beverage clarity by breaking down polysaccharide–protein complexes [145,146,147] The main challenge in using this method stems from the heavy-duty processing conditions during juice clarification, particularly the elevated temperatures and low pH, which may compromise enzymatic activity [145]. Nevertheless, the application of immobilized enzymes, as demonstrated in wine production, may be a valuable direction for future research aimed at enhancing the stability of sugar-sweetened beverages [146]. Immobilized pectinases have demonstrated good operational stability under conditions simulating industrial processes, temperatures of 50–60 °C, and pH levels of 3.5–4.0, which validates their potential applicability in beverage production processes [145,146].

Efficient flocculation inhibition can be achieved in various ways, e.g., through a combination of biopolymer stabilization, electrostatic control, adsorption of reactive forms, and potentially enzymatic treatments. The strategies based on natural materials offer an eco-friendly complement to impurity-reduction approaches.

### 8.3. Control of Beverage Production Processes

Effective control over beverage production processes is essential to minimize ABF formation. Critical process parameters—pH, temperature, and ingredient composition and quality—must be carefully controlled to maintain beverage stability and quality.

Firstly, the pH of beverages significantly impacts electrostatic interactions between proteins, polysaccharides, and polyphenols, influencing flocculation processes. In model beer and wine systems, maximum haze was observed between pH 4.0 and 5.5, depending on the alcohol content; at acidic or neutral pH values, haze intensity decreased [31,66]. Temperature during processing and storage strongly influences aggregation kinetics. Elevated temperatures (>50–60 °C) result in the denaturation of haze-active proteins and accelerate their cross-linking with polysaccharides or polyphenols, promoting visible flocculation. Processing and storage below 20 °C markedly slow these reactions and help to maintain beverage clarity [65,148,149,150]. Formulation and ingredient selection are equally important for floc control. Stabilizing agents such as selected hydrocolloids (e.g., depolymerized pectin, gum arabic) can help prevent flocculation by providing steric stabilization and limiting coacervate formation. Hydrocolloids form protective colloidal barriers around macromolecules, significantly enhancing beverage stability [151]. Additionally, antioxidants (e.g., ascorbic acid) and chelating acids (e.g., citric acid) are widely used in fruit juices and soft drinks to slow oxidative reactions and enzymatic browning by scavenging oxygen and complexing transition metals. By moderating polyphenol oxidation pathways, these additives may also influence the evolution of protein–polyphenol complexes and haze during storage, although direct evidence for a systematic reduction in protein–polyphenol flocs in sugar-sweetened beverages remains limited [31,66,152]. The main process levels, key interventions and their expected impact on ABF/floc risk are summarized in Table 3.

Minimizing acid beverage floc formation in sugar-sweetened beverages requires a comprehensive, multi-level strategy combining actions at both the sugar production and beverage formulation stages. The first line of defense lies in effectively reducing sugar impurities through careful selection of raw materials, optimized clarification and filtration processes, and the application of sustainable and innovative purification methods. In parallel, the strategic use of flocculation inhibitors, biopolymers, electrostatic stabilizers, advanced adsorbents, and emerging enzymatic treatments can further enhance beverage stability by disrupting or preventing aggregation phenomena. Finally, precise control over beverage production processes, including pH, temperature, real-time analytical monitoring, and ingredient assortment offers essential tools for maintaining clarity of beverages and consumer acceptance. Integrating these strategies holistically enables manufacturers to meet the quality demands of the beverage market.

## 9. Conclusions and Future Directions

Visible flocs in sugar-sweetened beverages arise mainly from protein and polyphenol interactions and colloidal instability—in alcoholic matrices, ethanol intensifies aggregation (alcohol flocs). These mechanisms have been consistently described across beverages and are relevant to refined sugars used as ingredients. Although ICUMSA ABF methods provide the operational definition of risk in acidic beverages, effective mitigation requires a two-tier approach integrating upstream removal of haze-active non-sugars in sugar/syrup processing and downstream stabilization of the finished beverage, which should be implemented with careful control of polyphenol removal so that beneficial phenolic compounds are not unnecessarily lost [6,31,60,66,155,156,157,158,159,160].

### 9.1. Key Conclusions

1. The primary driver of floc formation in sugar-sweetened beverages is the electrostatic interaction between oppositely charged colloidal particles at low pH. In alcoholic beverages, ethanol reduces colloidal stability and accelerates aggregate growth. These mechanisms are well-established in principle, but their relative importance in a given beverage depends on the impurity profile of the sugar used, which varies with the origin of raw material and processing conditions. The evidence linking specific impurities to ABF formation remains inconsistent across studies, and the dominant precursor cannot yet be predicted from sugar composition alone. This is the most important unresolved problem in the field.
2. Effective risk management requires a two-level strategy. At the sugar production stage, strict raw material quality control combined with optimized clarification, membrane filtration, and adsorptive purification reduces the load of haze-active proteins, polysaccharides, and silica carried in white sugar. At the beverage formulation stage, stabilizing agents, fining treatments, and careful control of pH, temperature, and ionic strength provide the second line of defense. Neither level alone is sufficient: upstream impurity reduction lowers but does not eliminate the risk, while downstream stabilization cannot compensate for poor sugar quality.
3. From an analytical perspective, the ICUMSA GS2-40 test remains the only validated reference for ABF risk assessment in white sugar, and all rapid or indirect predictors must be calibrated against it before industrial use. Colorimetric and spectrophotometric assays offer practical screening tools for individual precursor classes, but their predictive value depends on matrix-specific validation. Light-scattering techniques and ζ–potential measurements are sensitive to pre-visual aggregation and are currently underutilized. NIR spectroscopy with chemometrics is a promising direction for at-line screening, but no validated models for ABF-relevant sugar matrices have been published to date. A critical limitation of the current evidence base is that most predictive data derive from model systems rather than from commercially produced beverages, and this gap between laboratory findings and industrial reality has not yet been bridged.

### 9.2. Research Gaps and Future Work

1. Despite decades of work, ABF and alcohol flocs are still too often attributed to impurities rather than quantifiable interaction connections. A mechanistic, molecular-scale description of how proteins, polyphenols, polysaccharides, and mineral phases jointly control nucleation, growth, restructuring, and the reversibility of aggregates across realistic pH and ethanol ranges remains lacking. Priority should be given to experimental designs that systematically map interaction stoichiometry (protein–polyphenol ratios), solvent quality (ethanol), and electrostatics (ionic strength) while extracting kinetic parameters that can be compared across studies and matrices [60,66].
2. There is high scientific potential in linking modern colloid characterization with predictive modeling in a way that is transferable across beverage types. Instead of treating ζ–potential or particle size distributions as standalone indicators, future work should build mechanism-aware multivariate models that fuse time-resolved particle sizing and scattering outputs, electrokinetic descriptors (ζ–potential), and spectroscopic fingerprints. In particular, IR spectroscopy (FT-NIR/MIR) paired with chemometrics should be developed as a hypothesis-driven approach: models should be trained against clearly defined “ground truth” endpoints (ABF outcome, haze/turbidity trajectories) and tested on independent materials to avoid overfitting to a single matrix or producer [161,162,163].
3. A major gap is the lack of kinetic datasets that allow aggregation to be modeled and not just observed. Future studies should report time-resolved trajectories (size distributions, turbidity, and scattering intensity) under controlled perturbations (pH shifts, ethanol, and salt additions), enabling parameterization of aggregation frameworks (Smoluchowski-type kinetics; reversible/irreversible pathways) and comparison of rate constants across systems. Such kinetic benchmarking would also clarify when membrane/adsorbent “polishing” changes only the precursor concentration versus when it changes the dominant pathway. Published UF/MF case studies motivate the need for this deeper pathway-level evaluation rather than performance-only reporting [155,164].
4. Another research direction with strong scientific value is the development of selective interaction modifiers that act on specific floc formation points (polyphenol-binding sites, proline-rich domains, and multivalent polyphenol structures) rather than indiscriminately removing broad classes of compounds. Next-generation PVPP-type materials should be assessed not only according to turbidity reduction but also based on binding thermodynamics, competitive adsorption, and structural consequences for remaining colloids. This requires mechanistic assays (competitive binding experiments, adsorption isotherms, and spectroscopic tracking of complexation) to demonstrate why a modified adsorbent performs better and under which compositional regimes it fails [156].
5. Regarding standardization to enable cumulative science and robust validation, progress is currently limited by heterogeneous endpoints and incomplete reporting. Wider and more consistent use of ICUMSA method identifiers, GS2-40 (10-day ABF, definitive), GS2-44 (24 h ABF for beet white sugar, process control), and GS2-17 (conductivity ash, not directly comparable to gravimetric ash), together with explicit reporting of haze/turbidity units and minimal metadata (pH, ethanol %, ionic strength, temperature profile, storage time, and agitation history), would substantially improve comparability across studies and production sites. Such harmonized performance measures could be used to develop reliable analytical methods based on spectroscopic measurements and chemometric analyses (FT-NIR/MIR with multivariate calibration). In this way, classical analytical methods would enable the development of modern and rapid spectroscopic methods for floc determination.

## Figures and Tables

**Table 1:** ABF components originating from beet sugar (●) and cane sugar (●).

References	Saponins	Oleanolic Acid	Pectins	Carbohydrates	Polysaccharides	Carbon	Fats	Silica	Silicates	Proteins	Starch	Lipids	Waxes	Phenols	Dextran	Fragments of Plant Tissues
[36]	●		●													
[37]	●			●		●	●	●								
[30]	●	●			●					●						
[35]	●									●						
[47]						●		●		●	●		●			
[50]					●			●		●						
[51]					●		●	●		●	●	●	●			
[52,53,54]					●				●	●						
[56]					●						●					
[30]					●					●	●				●	
[4]												●	●			
[57]											●				●	
[49]			●		●			●		●	●	●		●		●
[5]					●				●	●	●					

**Table 2:** Comparative overview of analytical methods for floc detection and characterization.

Method	Analytical Target	Main Strengths	Main Limitations	Role in ABF/ Floc Studies
ICUMSA GS2-40 (10-day ABF test) [4,6,51]	Visible floc in acidified sugar solution; practical ABF endpoint.	Official reference method; direct industrial relevance; clear benchmark for validation.	Time consuming; partly subjective; poor sensitivity to early collision.	Reference assay for final confirmation of ABF tendency in white sugar solution.
ICUMSA GS2-44/ 24 h tests [16,35,85]	Visible floc tendency in beet white sugar.	Faster than ICUMSA GS2-40; useful for factory screening.	Lower standardization and inter-laboratory comparability.	Routine screening and process control, with positives interpreted against ICUMSA GS2-40.
Alcohol floc test [57,60]	Alcohol-induced precipitation of dextran, starch, levan and related polysaccharides.	Simple and fast; useful for alcoholic beverages; quantitative NTU threshold possible.	Specific to alcohol-floc context; not directly transferable to acid beverage flocs.	Targeted assessment of alcohol floc risk in sugar destined for use in alcoholic beverages.
Light/optical microscopy [49,87]	Macroscopic and microscopic morphology of flocs and entrapped plant fragments.	Simple visual confirmation of heterogeneity, the presence of tissue debris, and floc structure.	Low chemical specificity; limited for microscope resolution and existing aggregates.	Morphological examination of isolated flocs and confirmation of plant-derived debris.
SEM-EDS [49]	Surface morphology and elemental composition of isolated flocs.	Combines structural and elemental evidence; useful for silica- or mineral-rich particles.	Requires dried material; destructive preparation; expensive; not routine.	Confirmatory characterization of isolated flocs, especially mineral/inorganic contributions.
UV-Vis/turbidimetry/ colorimetric assays [3,57,61,92,110,111]	Proteins; phenolics; starch; dextran; turbidity and haze development.	Fast, accessible and quantitative; good for comparative screening and routine monitoring.	Usually indirect and matrix-sensitive; limited molecular specificity unless separately validated.	Rapid screening of haze intensity and selected precursor groups in routine workflows.
FTIR/ATR-FTIR and NIR/FT-NIR with chemometrics [3,114]	Broad spectral information related to polysaccharides, proteins, phenolics, mineral phases, overall composition, and instability risk.	Rapid and non-destructive analysis with minimal sample preparation. Useful for comparative analyses of IR spectra; attractive for routine industrial screening after calibration.	Both approaches are indirect for floc prediction. FTIR may be limited by band overlap and low trace level specificity. NIR requires robust calibration and external validation and still has limited ABF-specific evidence.	Complementary IR spectra of flocs, juices, and isolated fractions, with NIR representing an emerging tool for predictive screening.
DLS/SALS [4,34,106]	Particle size distribution, hydrodynamic diameter and early aggregation kinetics.	Sensitive to pre-visible colloidal growth; valuable for mechanistic and time-resolved studies.	Interpretation can be difficult in complex, polydisperse matrices; limited chemical specificity.	Early warning and mechanistic tracking of aggregation before visible flocs appear.
ζ–potential [4,34,115]	Surface charge and electrokinetic stability of colloidal particles.	Mechanisms associated with pH and ionic-strength-driven destabilization.	Indirect method; does not identify chemical compounds; strongly condition-dependent.	Best paired with light scattering to explain why dispersions destabilize.
HPLC/LC-MS/GC-MS [30,44,49,51,60,89,90,92,99,107]	Specific precursors and markers such as amino acids, phenolics, saponins and sugars.	High chemical specificity; strong indirect confirmatory; helps explain for floc formation hypotheses.	High cost, sample preparation burden and analytical complexity; less suited for routine screening.	Confirmatory identification and quantification of targeted floc-active compounds.
ELISA/XRD [87,96,97,114]	Dextran by immunoassay; aggregate crystal structure by diffraction.	High specificity for selected targets; useful when dextran or mineral phases are suspected.	Limited number of matrices; more target-specific than global floc assessment.	Problem-driven confirmatory analysis of suspected dextran contamination or inorganic compounds.

The abbreviations used in Table 2 are defined in the Abbreviations section.

**Table 3:** Process-level strategies to reduce floc risk in sugar-sweetened beverages.

Process Level	Key Strategy/Intervention	Expected Impact on ABF/Floc Risk	References
Raw material and sugar production	Strict raw material quality control (clean, undamaged beets/cane; monitoring of K, Na, α-amino N, dextran) and optimized purification process.	Lower initial load of plant-derived colloids and non-sugars entering the process; reduced turbidity and color in juices; fewer haze-active precursors carried into white sugar and syrups.	[49,116,117,118,119]
Membrane and adsorptive purification of juices and syrups	Microfiltration/ultrafiltration (ceramic or stainless-steel membranes), sometimes combined with activated carbon pretreatment or ion-exchange/adsorptive resins.	Effectively reduces turbidity, color and macromolecular impurities; removal of charged colorants and some inorganic non-sugars; lower ABF precursor load in liquid sugars and syrups.	[120,121,122,123,128]
Bio-based and sustainable purification	Application of anionic bioflocculants derived from sugarcane bagasse or pulp-based materials, as well as modified sugar beet pulp or chitosan-cellulose composites.	Clarification and color/turbidity reduction comparable to synthetic polymers, with improved biodegradability and alignment with circular bioeconomy concepts.	[125,127,129,130]
Beverage formulation	Use of stabilizing hydrocolloids (gum arabic, depolymerized pectin) and carefully selected fining agents (PVPP, silica gel, bentonite) adapted from beer and wine practice.	Steric and electrostatic stabilization of dispersions; targeted removal of haze-active protein and polyphenol fractions; lower haze and fewer visible flocs during shelf-life.	[31,65,67,133,137,144,151]
Beverage process control	Control of pH, temperature, ionic strength (especially Ca^2+^/Mg^2+^), and oxygen exposure during processing and storage; use of ICUMSA ABF tests (GS2-40/GS2-44) alongside haze/color metrics, with potential support from process analytical technology tools (FT-NIR/MIR).	Aggregation kinetics moderated; avoidance of conditions that maximize protein–polyphenol haze; early identification of high-risk lots and process drifts; more robust ABF risk control.	[6,16,31,65,66,121,134,135,136,153,154]

The abbreviations used in Table 3 are defined in the Abbreviations section.

## Data Availability

No new data were created or analyzed in this study. Data sharing is not applicable to this article.

## Supplementary Materials

The following supporting information can be downloaded at: https://www.mdpi.com/article/10.3390/molecules31081246/s1, Supplement S1: Search strategies, Supplement S2: Table S1. Expanded comparative overview of analytical methods used to detect and characterize floc precursors and flocs in sweetened beverages.

## Abbreviations

ABF	Acid beverage floc
ABV	Alcohol by volume
AFM	Atomic force microscopy
ATR	Attenuated total reflectance
ATR-FTIR	Attenuated total reflectance Fourier transform infrared spectroscopy
DLS	Dynamic light scattering
DLVO	Derjaguin–Landau–Verwey–Overbeek
dp	Date of publication
DS	Dry substance
EDS	Energy-dispersive spectroscopy
ELISA	Enzyme-linked immunosorbent assay
ELS	Electrophoretic light scattering
ESI	Electrospray Ionization
EtOH	Ethanol
FSTA	Food Science and Technology Abstracts
FT-IR	Fourier transform infrared
FT-NIR	Fourier transform near-infrared
GAE	Gallic acid equivalents
GC-MS	Gas chromatography-mass spectrometry
HPLC	High-performance liquid chromatography
IBP	Indigenous beet polysaccharide
IC-PAD	Ion chromatography with pulsed amperometric detection
ICUMSA	International Commission for Uniform Methods of Sugar Analysis
IR	Infrared
ISP	Indigenous sugar cane polysaccharide
LC	Liquid chromatography
LC-MS	Liquid chromatography–mass spectrometry
LD	Laser diffraction
LOD	Limit of detection
LOQ	Limit of quantification
MALDI-MS	Matrix-assisted laser desorption/ionization mass spectrometry
MF	Microfiltration
MIR	Mid-Infrared
MS	Mass spectrometry
NEAR/x	Terms within x words, any order
NIR	Near-infrared
NTA	Nanoparticle tracking analysis
NTUs	Nephelometric turbidity units
Ps	Proteins
PAT	Process analytical technology
PCC	Population, concept, context
PCS	Photon correlation spectroscopy
PET	Polyethylene terephthalate
pI	Isoelectric point
PPs	Polyphenols
PRE/n	First term precedes the second within n words
PRISMA	Preferred Reporting Items for Systematic reviews and Meta-Analyses
PRISMA-S	Preferred Reporting Items for Systematic reviews and Meta-Analyses—Search extension
PRISMA-ScR	Preferred Reporting Items for Systematic reviews and Meta-Analyses extension for Scoping Reviews
PVPP	Polyvinylpolypyrrolidone
Q-ToF	Quadrupole time-of-flight
QC	Quality control
SALS	Small-angle light scattering
SEM	Scanning electron microscopy
SLS	Static light scattering
SPE	Solid phase extraction
SPE–HPLC	Solid phase extraction–high-performance liquid chromatography
TLC	Thin-layer chromatography
TMB	Tetramethylbenzidene
TMS	Trimethylsilyl
TS	Topic search
UF	Ultrafiltration
UV	Ultraviolet
UV-VIS	Ultraviolet–visible
W/n	Within n words, any order
WoS	Web of Science
XRD	X-ray powder diffraction
