# Supplementary material for: Chemical Precursors of Flocs in Sweetened Beverages: Mechanisms of Formation, Analytical Methods, and Industrial Strategies
Source: Molecules. 2026 Apr 9;31(8):1246. doi: 10.3390/molecules31081246 (PMC13118384; doi:10.3390/molecules31081246)
Supplement: Supplementary file 1 [file molecules-31-01246-s001.zip › Supplement S2 Table S1.pdf]

Table S1. Expanded comparative overview of analytical methods used to detect and characterize floc precursors and flocs in sweetened beverages.

Method / technique	What It Detects	Sensitivity / LOD [*]	Main Strengths	Main Limitations	Recommended Use-Case
		**A. Floc Susceptibility Tests**			
ICUMSA GS2-40 (10-day ABF test) [4,6,51]	visible floc in acidified white sugar solution (pH 1.5, 10 days, ~25 °C); pass/fail endpoint	**[A]** flocs >~100 µm correlate with positive visual result (SALS-corroborated [4]); no numerical LOD defined in the standard	official international reference method; simple pass/fail interpretation; benchmark for all alternative methods	10-day duration; subjective visual assessment; unable to detect early-stage colloidal aggregation	benchmark reference assay for ABF tendency in white sugar; all alternative methods must be validated against GS2-40 before use
ICUMSA GS2-44 (24-h ABF test, beet sugar) [16,35]	visible floc in acidified beet white sugar solution (30%, pH 2.0, 24 h); pass/fail endpoint	**[–]** visual endpoint only; no numerical LOD defined in the standard	faster than GS2-40; suited for routine process control in beet sugar production	beet sugar only; less definitive than GS2-40; subjective visual endpoint	rapid process-control screening in beet sugar production; confirmation positive results with GS2-40 recommended
Modified ABF tests (CTC-LA-MT1-025; Carter & Jensen test; 24-h test variant) [35,57,85]	ABF in matrices with CO_2, sodium benzoate, visual assessment; spectrophotometric endpoint	**[A]** Carter & Jensen: higher sensitivity than GS2-40 [85]; 24-h test variant: turbidity at 400 nm, incubation ≥48 h recommended [35]; no numerical LOD published for any variant	closer to real beverage conditions; spectrophotometric variants reduce subjectivity	not standardized; limited inter-laboratory comparability; results not comparable across variants	when GS2-40 lacks sensitivity or realistic matrix representation
Alcohol floc test (60% ABV, NTU; CTC-LA-MT1-017) [57,60]	Insoluble polysaccharides (60% v/v EtOH) [60]; turbidity as endpoint	**[A]** Positive threshold: NTU ≥2.0 within 30 min [60]; absorbance at 420 nm (CTC-LA-MT1-017 version).	rapid (30 min); quantitative threshold criterion; directly applicable to invert sugar QC	alcohol-induced flocculation only; does not identify individual precursors; CTC method not internationally standardized	QC of sugar and invert sugar for alcoholic beverages; rapid batch suitability decision
		**B. Microscopy**			
Light / Optical Microscopy [49]	macro-floc morphology; plant tissue fragments (parenchymal cells, xylem, stomata)	**[T]** spatial resolution ~0.2 µm (Abbe diffraction limit); qualitative only; **[–]** no quantitative LOD defined in ABF research	simple; reveals tissue fragments and floc heterogeneity; reflects consumer-visible morphology	qualitative; no chemical information; ineffective for nanoscale aggregates	morphological screening; confirming biological debris in isolated floc

Method / technique	What It Detects	Sensitivity / LOD [*]	Main Strengths	Main Limitations	Recommended Use-Case
SEM/EDS [49]	elemental composition (Si)	**[T]** SEM spatial resolution: ~1–5 nm; EDS LOD: ~0.1–1 wt% (matrix-dependent); detects elements Z ≥ 4.	elemental data in a single measurement; definitive silica/silicate identification	requires dried and coated samples; destructive; high cost; not for routine use	verifying mineral and silicate contribution to floc composition
**C. Spectroscopic and Colorimetric Methods**					
UV-VIS Spectrophotometry (Amido Black 10B; 230/260 nm; color index) [84,91,92]	proteins (Amido Black 10B 630 nm; 230/260 nm absorbance); polyphenolic and flavonoid pigments via color index	**[A]** 230/260 nm method [94]: LOQ 1.5 mg/kg protein, linear range 1.5–16 mg/kg (25% DS) — values not independently verified from open-access sources. Amido Black 10B [91]: floc-positive 0,3-0,4% protein in sugar, floc negative 0,004-0,006% protein in sugar. Color index: absorbance ratio at 420 nm (pH 9/pH 3) [84].	fast, inexpensive, accessible; protein assays validated in refined sugar matrices; color index links pigment load to ABF risk	on-specific unless tied to a validated assay; matrix interference from colored extracts; no individual compound identification	Routine protein screening in refined sugar; color index as a proxy ABF risk marker; calibrate against GS2-40 before use as a predictor
Nephelometry (ISO 7027 compliant; 860 nm; 90° angle) [3,57,60,103,105]	bulk turbidity and intensity scattered light beam from suspended particles and aggregates (NTU)	**[T]** nephelometer resolution ~0.01 NTU (instrument-dependent; not specified in ISO 7027 itself; 860 nm, 90° detection). **[A]** ~1000-fold NTU increase upon floc formation in 60% ABV alcohol solution of invert sugar [60]; positive alcohol flocculation test NTU ≥ 2.0 [60]	rapid, quantitative; nephelometry highly sensitive for insoluble complexes at relatively low particle concentrations; useful for monitoring instability over time	non-specific; signal depends on particle size and matrix; no chemical identification	monitoring haze development between sugar lots; complement to ICUMSA tests; nephelometry preferred at low particle concentrations
FTIR / ATR-FTIR Spectroscopy [3,114]	functional groups of polysaccharides, proteins, phenolics, starch, Na^+ ions, SiO_2, phosphate ion concentrations; IR spectra (chemical fingerprint) of flocs	**[T]** FTIR **s**pectral resolution ≤4 cm^{-1} (measurement range 400–4000 cm^{-1}); **[A]** ATR-FTIR spectra 3800–2600 cm^{-1} and 1650–650 cm^{-1}). **[–]** no published LOD for ABF-specific analytes	rapid IR spectra with minimal reagents; identifies dominant chemical classes; integrates well with chemometrics	overlapping bands limit specificity; typically requires dried floc material; insufficient alone for trace component identification	structural characterization of isolated floc samples; IR spectra with chemometrics; ATR-FTIR combined with XRD and SALS for inorganic compounds
NIR / FT-NIR spectroscopy with chemometrics no ABF-specific	carbohydrates, proteins via overtone/combination bands; chemometric	**[–]** no published LOD for ABF-specific analytes; quantitative performance requires matrix-specific chemometric validation	fast, non-destructive, minimal sample preparation; strong	requires matrix-specific calibration and robust validation; for ABF no validated	emerging tool for predictive screening in sugar production; requires validation against ICUMSA

Method / technique	What It Detects	Sensitivity / LOD [*]	Main Strengths	Main Limitations	Recommended Use-Case
studies identified in this review	prediction of composition and ABF risk		potential for at-line or online PAT applications	models published; risk of overfitting	benchmarks before industrial use
Colorimetric assay: Bradford; Folin-Ciocalteu; ICUMSA GS1-16 (starch); heptamolybdate [3,5,89, 110,112,113]	protein (Bradford); total phenolics content as GAE/kg of sample (Folin-Ciocalteu); soluble starch (GS1-16, 700 nm); reactive and colloidal silicates (heptamolybdate method)	**[T]** Bradford: LOD ~5–6 µg protein/mL; linear range 125–1000 µg/mL (standard assay) or 1.25–25 µg/mL (microassay — values per commercial protocols; these ranges are not stated in the original Bradford (1976) paper); Folin-Ciocalteu: LOD ~0.2–1 mg GAE/L; linear range 50–500 mg GAE/L (standard cuvette-based method per Singleton & Rossi 1965 and Waterhouse/UC Davis protocol) ; ICUMSA GS1-16 (measured at 700 nm): validated working range 0–300 mg starch/kg sugar (raw sugar only); LOD in refined sugar matrices not established; heptamolybdate: LOD ~0.02 mg SiO_2/L (50-mL Nessler tubes) to 0.05 mg SiO_2/L (1-cm cell at 815 nm), water matrices; validated for the molybdosilicate blue (reduced) variant only	simple and inexpensive; covers four key precursor classes in a complementary panel; implementable in standard laboratory settings	non-selective (Bradford responds to all proteins; Folin-Ciocalteu to all reducing agents); GS1-16 excludes insoluble starch; heptamolybdate differentiates only reactive vs. colloidal silica	multi-parameter ABF precursor screening in refined sugar QC; correlate results with GS2-40 to establish predictive thresholds
**D. Particle Sizing and Colloidal Characterization**					
DLS / SALS [4,34,106]	hydrodynamic diameter distribution; floc growth kinetics; early aggregation below visual threshold; fractal dimension (SALS)	**[T]** DLS: size range ~0.3 nm–10 µm. **[A]** SALS: flocs >~100 µm correlate with positive visual ABF result, detects pre-visual colloidal aggregates below the visual threshold [4]	sensitive to pre-visual aggregation; non-destructive; enables time-resolved kinetic tracking; currently underutilized in ABF research	DLS skewed by large aggregates; polydisperse matrices difficult to interpret; no chemical identification; careful dilution required	complement to zeta potential and FTIR; early warning before GS2-40 threshold

Method / technique	What It Detects	Sensitivity / LOD [*]	Main Strengths	Main Limitations	Recommended Use-Case
Laser diffraction (LD) [3,106]	particle size distribution	**[A]** particle size range: 0.02–2600 μm	broad size range covering fine colloids through macro-aggregates; reproducible; no dilution required	no chemical identification; spherical particle assumption introduces error for non-spherical aggregates	particle size distribution of sugarcane juices and model systems; assessment of clarification efficiency
Zeta potential (z-potential) [4,34,115]	surface charge of colloidal particles as an indicator of electrokinetic stability	**[T]** measurement precision ±1–2 mV (typical ELS instruments); $\lvert\zeta\rvert$ >30 mV indicates colloidally stable dispersion (general colloidal criterion)	explains pH- and ionic-strength-driven destabilization; confirmed negative charge of silica and polysaccharide molecules	does not identify particle chemistry; affected by ionic strength, pH, and matrix; requires dilute suspensions	used alongside DLS/SALS to explain colloidal instability; monitoring the effect of pH and ionic strength on ABF precursors' charge, clarification efficiency
**E. Chromatographic and Immunochemical Methods**					
HPLC (SPE, IC-PAD) [60,92,99,107]	amino acids (post-hydrolysis); saponins (SPE-HPLC); oligosaccharides (IC-PAD); broad organic analyte range	**[T]** UV detection: ~0.1–1 μg/mL; fluorescence: ~1–10 ng/mL; amino acids (post-column): ~1–10 pmol injected; **[A]** Saponins (SPE-HPLC): low μg/kg range	highly versatile; excellent reproducibility; broadest analyte range of any single platform	requires sample preparation and method development per analyte class; high cost; not for routine factory use	confirmatory analysis of floc proteins, saponins, and oligosaccharides; resolving conflicting data on precursor identity
LC-MS / LC-UV-MS Q-ToF; MALDI-MS [44,49,89,90]	phenolic compound; flavones; saponin aglycones; fatty acids and waxes in ABF (Q-ToF/MALDI-MS)	**[T]** ESI-MS/MS: ~0.1–10 μg/kg (general value for phenolics); Q-ToF: mass accuracy <5 ppm, LOD ~0.1–10 ng/mL; MALDI-MS: ~1–100 fmol (analyte-dependent). Note: Colombo et al. [90] is a qualitative identification study — no LOD was reported	highest chemical specificity; structural identification of unknowns; Q-ToF/MALDI-MS enables non-targeted profiling of novel floc components	high cost and complexity; matrix effects in sugar solutions; unsuitable for routine QC	structural identification of phenolic, saponin, and lipid precursors; non-targeted profiling of unknown floc components
GC-MS (with derivatization) [30,51]	saponin aglycones (oleanolic acid) in beet extracts (TLC and GC-MS) [30]; monosaccharide composition of	**[T]** monosaccharides as alditol acetates: ~0.1–1 μg/mL; saponin aglycones (TMS derivatives): ~10–100 ng/mL; **[–]** no published LOD for ABF applications	high sensitivity for derivatizable analytes; definitive monosaccharide composition; large reference spectral libraries	derivatization required for polar/high-MW analytes; not applicable to intact	monosaccharide composition of floc polysaccharide fractions; saponin aglycone identification in beet sugar extracts

Method / technique	What It Detects	Sensitivity / LOD [*]	Main Strengths	Main Limitations	Recommended Use-Case
	polysaccharide fractions after hydrolysis			polysaccharides or proteins	
ELISA (sandwich; dextran-specific) [96]	dextran in raw sugar and sugar by-products	**[A]** LOD: 3.9 ng/mL; working range: 7.8–500 ng/mL [96]; validated in sugar matrices with 96.35–102.00% spike recovery	high sensitivity and specificity; faster than enzymatic or chromatographic dextran methods; kit format for factory implementation, high accuracy, reliability, short analysis time	targets dextran only; antibodies or commercial kits required; not a global ABF assay, lack of sensitivity to low-molecular-weight dextran (<10^5 kDa) and low level (< 0.2 g/kg on solids)	targeted dextran QC in raw and refined cane sugar when dextran is suspected as a primary ABF driver
**F. Structural and Diffraction Methods**					
X-ray powder diffraction (XRD) [3, 87,114]	aggregate crystal structure, size and shape of calcium phosphate flocs in sugar juices, silica	**[T]** phase detection LOD: ~1–3 wt% (conventional XRD); crystallite size ~5–100 nm (Scherrer equation); quantitative phase analysis by Rietveld refinement; **[–]** no published LOD for ABF-specific inorganic phases	crystal structure identification; quantitative composition possible; combines well with ATR-FTIR and SALS	amorphous components undetected; requires dried isolated samples; not applicable to organic floc components	characterization of flocs from juice clarification

[*] Where numerical LOD values for ABF-relevant sugar matrices are unavailable, the entry describes the instrumental measurement range or practical detection threshold reported for comparable matrices; **[A]**: ABF / sugar-specific value from floc literature; **[T]**: Technical specification of instrument or standardized method; **[–]**: No published data available for sugar / beverage matrices

The abbreviations used in Table S1 are defined in the Abbreviations section.
