# Supplementary material for: Chemical Precursors of Flocs in Sweetened Beverages: Mechanisms of Formation, Analytical Methods, and Industrial Strategies
Source: Molecules. 2026 Apr 9;31(8):1246. doi: 10.3390/molecules31081246 (PMC13118384; doi:10.3390/molecules31081246)
Supplement: Supplementary file 1 [file molecules-31-01246-s001.zip › Supplement S1 Search strategies.pdf]

Supplement S1 Search strategies, run metadata, and PRISMA counts

Interfaces used: Scopus (Elsevier), Web of Science Core Collection (Clarivate), PubMed; FSTA via [EBSCO/Ovid]; CAB Abstracts via [CABI/EBSCO/Ovid]; run date/time: 25 October 2025, Europe/Warsaw. Limits: 2000–2025 (inclusive). Language: English (primary; non-EN screened if methods/results extractable). Documentation standard: PRISMA-S.

PubMed (Title/Abstract focus)

```
("acid beverage floc"[tiab] OR "alcohol floc"[tiab] OR
(beverage*[tiab] AND (floc*[tiab] OR haze[tiab] OR
turbidity[tiab])) OR (protein*[tiab] AND polyphenol*[tiab] AND
(complex*[tiab] OR interact*[tiab]))) AND (sugar[tiab] OR
sucrose[tiab] OR "white sugar"[tiab] OR "refined sugar"[tiab])
AND ("2000/01/01"[dp] : "2025/09/23"[dp])
```

Returned: 96 records.

Scopus (Advanced; TITLE-ABS-KEY)

```
TITLE-ABS-KEY(("acid beverage floc" OR "alcohol floc" OR
(beverag* W/3 (floc* OR haze OR turbidity)) OR ((protein* W/3
polyphenol*) AND (complex* OR interact*))) AND (sugar OR
sucrose OR "white sugar" OR "refined sugar")) AND (PUBYEAR >
1999 AND PUBYEAR < 2026)
```

Returned: 412 records. (Operator semantics W/n, PRE/n.)

Web of Science Core Collection (Topic/TS)

```
TS=(("acid beverage floc" OR "alcohol floc" OR (beverag*
NEAR/3 (floc* OR haze OR turbidity)) OR ((protein* NEAR/3
polyphenol*) AND (complex* OR interact*))) AND (sugar OR
sucrose OR "white sugar" OR "refined sugar"))
```

Timespan 2000–2025; Indexes: SCI-EXPANDED, ESCI. Returned: 298 records. (Operator NEAR/x.)

FSTA

Logic replicated (Topic/Abstract fields as supported). Returned: 134 records. Database scope documented by IFIS.

CAB Abstracts

Logic replicated (Topic/Abstract fields as supported). Returned: 88 records. Database scope documented by CABI.

ICUMSA / grey sources

Targeted retrieval of GS2-40 (2019), GS2-44 (2019), GS2-17 (2011) and related site pages; non-indexed items: 9 records.
